# Systematic evaluation of TCGA tumor microbiota reveals context-dependent reliability

**DOI:** 10.1128/msystems.00180-26

**Published:** 2026-04-22

**Authors:** Chenchen Ma, Changxing Su, Jiaxuan Li, Jiaying Wang, Jianliang Liao, Lanlan Cheng, Jiuxin Qu, Guoquan Zhang, Jun Jiang, Shimin Shuai

**Affiliations:** 1 Department of Human Cell Biology and Genetics, School of Medicine, Southern University of Science and Technology 639321, Shenzhen, Guangdong, China; 2 SUSTech Homeostatic Medicine Institute, School of Medicine, Southern University of Science and Technology 639321, Shenzhen, Guangdong, China; 3 Department of Clinical Laboratory, Shenzhen Third People’s Hospital, National Clinical Research Center for Infectious Diseases, The Second Affiliated Hospital of Southern University of Science and Technology 535206, Shenzhen, Guangdong, China; 4 Medical Center of Stomatology, Shenzhen People's Hospital (The First Affiliated Hospital of the Southern University of Science and Technology and The Second Clinical Medical College of Jinan University) 255310, Shenzhen, Guangdong, China; 5 Department of Emergency Medicine, The First People’s Hospital of Foshan (Foshan Hospital Affiliated to Southern University of Science and Technology), School of Medicine, Southern University of Science and Technology, Foshan, Guangdong, China; University of Pittsburgh Medical Center, Pittsburgh, Pennsylvania, USA

**Keywords:** tumor microbiome, host-microbe interaction, pan-cancer analysis, benchmarking, TCGA

## Abstract

Microbial profiles from The Cancer Genome Atlas (TCGA) are widely used to study the tumor microbiota, a key component of the cancer ecosystem, yet their reliability remains unclear. Here, we systematically benchmarked two leading TCGA microbial profiles (TMPs) to define their consistency, accuracy, and reliability in host-microbe association studies across 24 cancer types, with a primary focus on the bacterial component. We found that while the TMPs showed substantial agreement in microbial composition, their accuracy in detecting known oncomicrobes was variable, ranging from excellent for human papillomavirus (HPV) to poor for *Helicobacter pylori*. The concordance of downstream host-microbe associations was moderate for gene expression but nearly absent for methylation and protein data. Our permutation-based framework revealed that while most individual associations were statistically reliable, those involving cell type composition and patient survival were statistically spurious. To empower future research with these insights, we introduced Multi-Omics and Microbiome Associations in Cancer 2 (MOMAC2), an interactive web portal that stratifies all associations by confidence level. We demonstrated its utility by using high-confidence associations to confirm HPV-driven methylation-gene expression axes and guide a novel experimental investigation. Co-culture with *Streptococcus anginosus* not only validated its predicted gene expression changes in oral cancer cells but also revealed a significant promotion of cancer cell proliferation and migration. Our study provides a rigorous framework for interpreting TCGA’s tumor microbiome and highlights that these data require careful, multi-layered validation to yield robust biological insights.

**IMPORTANCE:**

Bacteria living inside tumors can influence how cancer grows and responds to treatment, but the field has been hampered by controversy over the reliability of the data. Our study provides a much-needed road map for researchers. We rigorously tested the massive Cancer Genome Atlas data set and developed a statistical framework to separate true biological signals from random noise. We discovered that many widely reported links are statistically unreliable and likely false leads. Importantly, our framework successfully pinpoints trustworthy signals. We used it to identify a specific bacterium, *Streptococcus anginosus*, and proved in the lab that it makes oral cancer cells grow faster and spread. Our publicly available Multi-Omics and Microbiome Associations in Cancer 2 (MOMAC2) web portal now allows scientists to use these reliability-graded findings to accelerate robust cancer microbiome research.

## INTRODUCTION

The intricate interplay between cancer and its microbial residents, collectively termed the tumor microbiota, is emerging as a pivotal force within the cancer ecosystem (1–5). Recent large-scale analyses have broadened our understanding, revealing bacteria, archaea, viruses, and fungi residing within tumors and metastases, potentially influencing oncogenesis (6, 7), progression (8, 9), tumor microenvironment (10, 11), therapeutic responses (12–15), and patient survival (15, 16).

Despite these compelling insights, the existence and functional significance of tumor microbiota remain subjects of intense scientific debate. A key challenge lies in the inherent difficulty of identifying low-biomass microbial communities from host genome sequencing data, often complicated by suboptimal bioinformatics approaches and insufficient contamination controls in earlier studies (4, 5, 17–20). The Cancer Genome Atlas (TCGA), a monumental cancer sequencing initiative, offers an unparalleled multi-omics resource (genome, transcriptome, proteome, and DNA methylome) for nearly 20,000 primary cancer and matched normal samples across 33 cancer types. This wealth of data has led to the development of numerous TCGA microbial profiles (TMPs) (2, 3, 5, 17, 21–24). However, the high false-positive rates in early TMPs have fueled significant controversy (5, 19, 20, 25). Disturbingly, these potentially error-prone early TMPs continue to be cited and utilized (26–29), casting doubt on the reliability of subsequent conclusions (30).

In response to these criticisms, more rigorous and updated TMPs have been developed, exemplified by recent contributions from Sepich-Poore et al. (5) and Ge et al. (17). These profiles incorporate improved host-read removal, updated reference databases, and more stringent contamination filtering. Nevertheless, the low microbial biomass in tumor tissues continues to pose analytical challenges, and the lack of a universally accepted computational pipeline inevitably leads to inconsistencies across studies. A comprehensive, head-to-head comparison of these representative and updated TMPs is, therefore, critically needed to clarify their consistency, strengths, and limitations, providing essential guidance for their appropriate use in tumor microbiota research.

In this study, we present a systematic comparison of these two independent TMPs across 24 cancer types. Our objectives were threefold: first, to assess their quantitative and qualitative consistency; second, to evaluate their accuracy against clinically validated microbial biomarkers; and third, to rigorously benchmark the reliability and concordance of host-microbe associations identified through multi-omics data using a permutation-based approach. To facilitate robust investigation, we also developed Multi-Omics and Microbiome Associations in Cancer 2 (MOMAC2) to provide confidence levels for host-microbe interactions. Our findings not only illuminate the current promise of utilizing TCGA-derived microbial profiles but also underscore the imperative for methodological rigor and cautious interpretation in this rapidly evolving field.

## RESULTS

### Substantial agreement in independent TCGA tumor microbiota profiles

To assess the reliability and generalizability of existing tumor microbiota analyses, we systematically compared two recently published and representative TCGA-derived microbial profiles by Sepich-Poore et al. (5) and Ge et al. (17). These profiles, both derived from whole-genome sequencing (WGS) data of primary tumor tissues, employed distinct methodologies for host-read depletion, microbiome profiling, and contamination removal. Sepich-Poore et al. (5) identified 583 microbial species from 1,835 samples across 24 cancer types, while Ge et al. (17) reported 11,895 species from 2,300 samples across the same 24 cancer types. Our integrated analysis focused on a total of 2,327 unique samples, with 1,808 samples overlapping between the two TMPs (Fig. S1A and B; Table S1).

To ensure robustness and minimize potential false positives, we applied stringent filtering criteria. We retained 231 species (225 bacteria, 3 viruses, and 3 fungi; Table S2) consistently identified in both TMPs. Additionally, we included species detected in only one data set but demonstrating a relative abundance exceeding 1% in at least one cancer type. This refined approach resulted in 268 microbial species (256 bacteria, 9 viruses, and 3 fungi) from Sepich-Poore et al. (5) and 352 species (299 bacteria, 14 viruses, and 39 fungi) from Ge et al. (17) for pan-cancer analyses (Fig. S1C; Table S2). Across cancer types, species richness (the total number of different species) varied considerably, from 3 to 243 in the Sepich-Poore et al. data set and from 33 to 345 in the Ge et al. data set (Fig. S1D). The number of shared species between the two TMPs within each cancer type ranged from 3 to 209, and notably, the species richness across cancer types showed a significant positive correlation between the two TMPs (Spearman’s rho = 0.93, *P* = 5.9 × 10^−7^, Fig. S1E).

**Fig 1:**
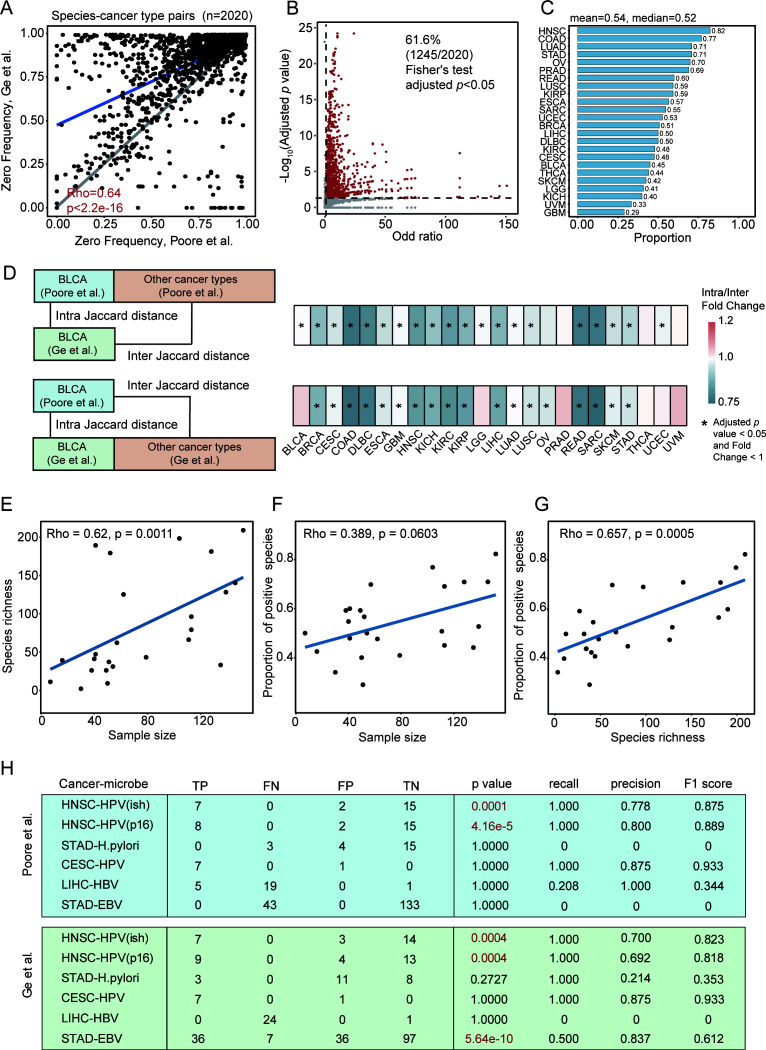
Two independent TCGA microbial profiles show substantial consistency but variable accuracy. (**A**) Spearman correlation of species- and cancer type-specific zero frequencies between the two TMPs. Each point represents a species-cancer type pair. The blue line represents the fitted trend, while the gray line shows the perfect correlation. (**B**) Consistency of binary presence/absence between TMPs, assessed by Fisher’s exact test. Red points represent species-cancer type pairs with significant associations (BH-adjusted *P* < 0.05), while gray points indicate non-significant ones. (**C**) Proportion of microbial species per cancer type showing significant concordance by Fisher’s exact test. (**D**) Comparison of intra-cancer vs inter-cancer Jaccard distances. The intra-cancer distance (comparing the same cancer type between TMPs, e.g., BLCA-Poore vs BLCA-Ge) is compared to the distribution of inter-cancer distances (e.g., BLCA-Poore vs all other cancers in Ge). *P* values were calculated using the Wilcoxon rank-sum test. Asterisks indicate significant differences (adjusted *P* < 0.05) where intra-distances are smaller than inter-distances. (**E–G**) Spearman correlations between (**E**) sample size and species richness, (**F**) sample size and concordance proportion (from panel C), and (**G**) species richness and concordance proportion. Each point represents a cancer type. (**H**) Performance metrics for detecting known oncomicrobes against clinical or orthogonal standards. Human papillomavirus (HPV) detection in head and neck squamous cell carcinoma (HNSC) relied on *in situ* hybridization or p16INK4a immunohistochemistry (P16). TP, true positive; TN, true negative; FP, false positive; and FN, false negative. Red font indicates a significant correlation with the gold standard by Fisher’s exact test.

Consistent with the low microbial biomass nature of cancer, both TMPs were highly sparse; at the pan-cancer level, 94.4% and 85.0% of all entries were zero, respectively, and the zero frequencies for each species within each cancer type were significantly correlated between the TMPs (rho = 0.64, *P* < 2.2 × 10^−16^; Fig. 1A; Tables S3 to S5). This high degree of data sparsity presents a statistical challenge for standard quantitative comparisons. We therefore prioritized assessing agreement on microbial presence or absence using Fisher’s exact test, which found a significant agreement for 61.6% (1,245 of 2,020) of all possible species-cancer type pairs (Benjamini-Hochberg [BH]-adjusted *P* < 0.05) (Fig. 1B; Table S6). Across individual cancer types, an average of 54% of microbial features demonstrated significant associations (Fig. 1C). To complement this, we assessed quantitative agreement for shared microbes with non-zero abundance in both TMPs. A Spearman’s correlation of abundance showed that 57.1% (540/945) of testable pairs had a significant positive correlation (BH-adjusted *P* < 0.05 and rho > 0), indicating good quantitative agreement (Fig. S2A and B; Table S7). Evaluation of Shannon and Simpson indices revealed significant correlations between TMPs in half of the cancer types (12/24) (Fig. S3; Table S8). Despite this correlation, systematic quantitative discrepancies emerged: Sepich-Poore et al. (5) reported significantly higher diversity scores in 11 (Shannon) and 13 (Simpson) cancer types, whereas Ge et al. (17) reported higher scores in only 5 and 3 types, respectively.

A key hypothesis in tumor microbiology is that different cancer types harbor distinct microbial signatures. To test whether these signatures are robust enough to be consistently identified across different analytical pipelines, we performed a Jaccard distance comparison using the binary infection status. For each cancer type in the Sepich-Poore et al. data set, we calculated its “intra-cancer” distance (comparing its profile to the corresponding profile from Ge et al.) and compared this to its “inter-cancer” distances (comparing its profile to all other cancer types from Ge et al.). A reciprocal comparison was also performed. Our analysis revealed that for the majority of cancer types (19 and 20 out of 24 for Sepich-Poore et al. and Ge et al., respectively), the intra-cancer distance was significantly lower than the inter-cancer distances (Fig. 1D; Fig. S4 and S5; Table S9). By applying the abundance-weighted Jensen-Shannon Divergence metric, we confirmed that intra-cancer distances were significantly lower than inter-cancer distances in 13 and 19 of the 24 cancer types for the Sepich-Poore et al. and Ge et al. data sets, respectively (Fig. S6 to S8; Table S10). This finding indicates that cancer-type-specific microbial signatures are robust and reproducible, demonstrating they are biological features rather than processing artifacts.

Finally, we explored potential technical and biological drivers of this consistency. We found that while sample size was significantly correlated (rho = 0.62, *P* = 0.0011; Fig. 1E) with the number of species detected (species richness), it was not significantly associated with the proportion of concordant microbes (rho = 0.389, *P* = 0.0603, Fig. 1F). Instead, species richness was significantly correlated with a higher proportion of concordant species as determined by the Fisher’s exact test (rho = 0.657, *P* = 0.0005; Fig. 1G). These results suggest that the reproducibility of microbial signatures is less a function of sample size and more a feature of the tumor’s underlying microbial diversity. Consequently, tumor types with lower species richness may present a greater challenge for cross-platform validation and warrant cautious interpretation.

Collectively, these findings demonstrate a notable degree of compositional consistency between these two independent TMPs despite their differences in analytic pipelines.

### Inconsistent oncomicrobe detection against clinical and orthogonal evidence

While the two TMPs show broad compositional agreement, their accuracy in identifying individual, clinically relevant oncomicrobes required direct evaluation. To assess their performance, we benchmarked both TMPs against established infection statuses. For human papillomavirus (HPV), *Helicobacter pylori* (*Hp*), and hepatitis B virus (HBV), we used the gold standard of clinically validated data from the same TCGA samples. For Epstein-Barr virus (EBV), we used an orthogonal standard derived from viral gene expression via RNA-Seq (Table S11).

Our benchmarking against clinical gold standards revealed a stark performance contrast. Both TMPs demonstrated high accuracy in detecting HPV (Fig. 1H). In head and neck squamous cell carcinoma (HNSC), F1 scores ranged from 0.82 to 0.89 (*n* = 23–26), while in cervical squamous cell carcinoma (CESC), the F1 score reached 0.93 (*n* = 7). Biologically, this was also supported by the significant overexpression of the HPV biomarker p16INK4A in the TMP-identified HPV-positive samples (Fig. S9; *P* = 2.0 × 10^−5^). In stark contrast, performance was poor for other oncomicrobes. Both TMPs demonstrated low accuracy for *Hp* in stomach adenocarcinoma (STAD), and both also failed to reliably detect HBV in liver hepatocellular carcinoma (LIHC). Critically, outside of HPV in HNSC, the concordance between TMP-derived status and the clinical gold standard was not statistically significant.

Next, we evaluated the TMPs against orthogonal EBV data from STAD (31). This comparison revealed a critical discrepancy: the Ge et al. profile identified EBV with high accuracy (F1 score = 0.61, Fisher’s exact test, *P* = 5.64 × 10^−10^), whereas the Sepich-Poore et al. profile failed to detect EBV in any STAD samples.

In summary, our validation against both clinical standards and orthogonal sequencing data demonstrates that while TMPs may agree on broad community structure, their accuracy for detecting specific oncomicrobes is highly variable. Performance ranges from excellent (HPV) to poor (*Hp* and HBV) and can be entirely pipeline-dependent, as highlighted by the conflicting results for EBV.

### Moderate and omics-dependent concordance in host-microbe associations

**Fig 2:**
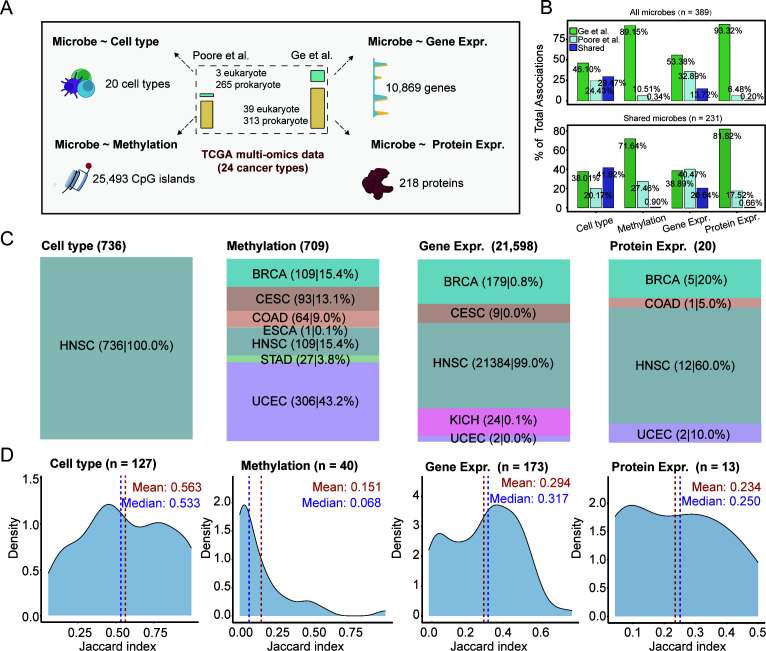
Host-microbe associations show moderate and omics-dependent concordance. (**A**) Schematic of the host-microbe association analysis, testing microbial presence/absence against four host multi-omics layers across 24 cancer types. Associations were assessed using Wilcoxon rank-sum tests. (**B**) Concordance of significant associations between the TMPs of Sepich-Poore et al. and Ge et al. TMPs. The *y*-axis shows the percentage of associations that are unique to Ge et al. (green), unique to Sepich-Poore et al. (light blue), or shared (dark blue). Concordance is shown for analyses using all microbes (top) and only the 231 shared microbes (bottom). (**C**) Distribution of shared host-microbe associations across cancer types, showing that concordance is driven by a few specific cancers. (**D**) Jaccard index distribution for associations involving shared microbes at the species level. The number (*N*) indicates the number of microbial species with at least one association.

A primary application of TMPs is to identify associations between tumor-resident microbes and host molecular features. We next investigated whether the compositional agreement between the two profiles extends to downstream association analyses. To this end, we tested for correlations between microbial presence/absence and four layers of host multi-omics data: cell type proportions (*n* = 20), CpG island methylation (*n* = 25,493), gene expression (*n* = 10,869), and protein expression (*n* = 218) across 24 cancer types, applying the Wilcoxon rank-sum test (Fig. 2A).

While each TMP individually yielded numerous significant associations (BH-adjusted *P* < 0.1; Table S12), the overlap between them was highly dependent on the molecular data layer (Fig. 2B). Concordance was highest for associations with cell type composition, with 736 shared associations found (Jaccard index [JI] = 0.29). This consistency improved when the analysis was restricted to the 231 shared microbes, increasing the JI to 0.42. The trend was similar for gene expression, with 21,598 shared associations (JI = 0.14, increasing to 0.21 for shared microbes). In stark contrast, the overlap was extremely poor for epigenomic and proteomic data, with only 709 shared methylation associations (JI = 0.0034) and a mere 20 shared protein associations (JI = 0.0021).

This moderate pan-cancer concordance was not uniform but was largely driven by a few cancer types with strong biological signals. For instance, the majority of shared cell-type and gene expression associations were concentrated in HNSC, while methylation associations were primarily found in UCEC, HNSC, and BRCA (Fig. 2C). This variability was also evident at the species level, where the median JI of associations per microbe ranged from a high of 0.53 for cell types to a low of 0.07 for CpG methylation (Fig. 2D; Table S13).

Collectively, these findings demonstrate that while individual TMPs can generate thousands of host-microbe associations, the consistency of these findings is moderate at best, contingent on the omics layer, underscoring the need for careful cross-validation. Given that the detected associations were predominantly driven by bacterial and viral signals, our subsequent analyses focused specifically on bacteria and viruses, rather than fungi.

### Contrasting reliability in host-microbe associations revealed by permutation benchmarking

**Fig 3:**
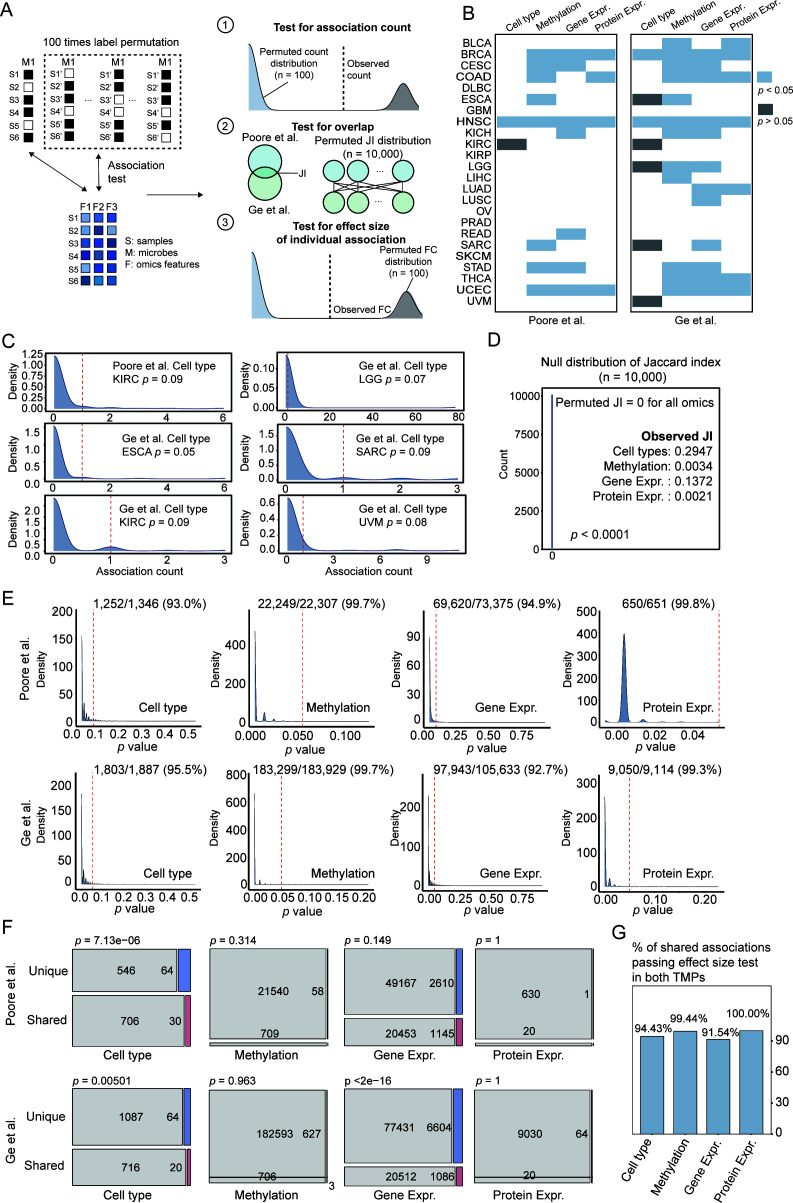
Permutation benchmarking reveals contrasting reliability of host-microbe associations. (**A**) Schematic of the permutation framework. Sample labels for microbial abundance were randomly shuffled 100 times to create empirical null distributions. The credibility of TMPs was assessed at three levels: association counts, association overlaps (Jaccard index), and association effect sizes (fold changes [FCs]). (**B**) Significance of the total number of associations per cancer type and omics layer. The *P*-value is the proportion of permutations yielding an equal or greater number of hits than the original data. Cancer-omics pairs with *P* > 0.05 suggest statistically spurious associations (dark gray). (**C**) A detailed view of the spurious microbe-cell type associations, where the number of hits in the original data (dashed line) is often within the null distribution from permutations (blue density). (**D**) Significance of the observed overlap. The JI of the real data is compared to the null distribution of JIs from permuted pairs, showing the observed overlap is highly non-random (*P* < 0.0001). (**E**) Distribution of effect size test *P*-values for individual associations across omics layers for both TMPs. Dashed red lines indicate the threshold for statistical significance. Numbers and percentages of significant association are shown in the figure. (**F**) Comparison of effect size test results for shared vs unique microbial associations by Chi-square tests. Gray represents associations that failed the test (*P* ≥ 0.05), while blue and red indicate significant negative and positive associations that passed the test (*P* < 0.05), respectively. (**G**) Percentage of shared associations that pass the permutation test for effect size in both TMPs.

To move beyond consistency and assess statistical robustness, we developed a permutation-based benchmarking framework to test whether our findings could be explained by random chance (Fig. 3A). For each TMP, we generated 100 permuted data sets by randomly shuffling sample labels while preserving the original microbial presence rates. These permuted data sets provided empirical null distributions to address a series of key questions regarding the data’s reliability. We first tested whether the total number of identified associations was significant, then assessed if the observed overlap between TMPs was non-random, and finally evaluated the reliability of the effect sizes for individual associations.

First, we tested whether the total number of significant associations found in the real data was greater than expected by chance. For CpG methylation, gene expression, and protein expression, the original TMPs consistently yielded a significantly higher number of associations than the permuted null distributions across nearly all cancer types (permuted *P* < 0.05; Fig. 3B; Table S14). However, we observed a striking exception for cell type composition, where the number of associations found in the original data was often statistically indistinguishable from random chance except for HNSC (Fig. 3B and C). This critical finding suggests that many microbe-cell type associations are likely spurious artifacts within the current statistical framework. To ensure the robustness of our findings, we conducted a sensitivity analysis by increasing the number of permutations to 1,000. The resulting *P*-values and significance classifications demonstrated high consistency with the initial results obtained from 100 permutations (Fig. S10A).

Second, we tested the significance of the overlap between the two TMPs. By comparing the Jaccard index of the real data to a null distribution generated from permuted pairs, we found virtually no overlap between permuted association sets (JI = 0, *P* < 0.0001; Fig. 3D). This provides strong statistical evidence that the observed concordance between the two TMPs, while moderate, is a genuine signal and not a product of random chance.

Finally, we used the framework to evaluate the reliability of individual association effect sizes. More than 92% of associations identified in both TMPs were highly statistically reliable, exhibiting effect sizes significantly greater than those from their corresponding null distributions (Fig. 3E). We also confirmed that associations shared between both TMPs were significantly more robust than associations unique to one TMP for cell types and gene expression (*P* < 0.05, chi-square test) (Fig. 3F). Note that shared cell-type associations were all from HNSC. Nevertheless, even among associations replicated across both TMPs, a fraction (e.g., up to 8.5% for gene expression) failed this stringent permutation test, underscoring the importance of evaluating each candidate association on its own statistical merits (Fig. 3G).

In summary, our permutation benchmark provides a robust statistical framework to distinguish statistically reliable host-microbe associations from random noise. It validates the overall significance and effect sizes of associations found for methylation, gene expression, and protein data, while simultaneously flagging the entire class of cell-type associations as highly unreliable within the current statistical framework.

### Variable validation success for host-gene associations in external data sets

To assess the biological relevance and generalizability of the TMP-derived findings, we sought to validate the identified host-gene expression associations using 26 independent, publicly available data sets. These external cohorts encompassed a range of experimental models, including *in vitro* co-cultures, naturally infected cell lines, and primary tumor tissues, providing a robust test of reproducibility (Table S15).

**Fig 4:**
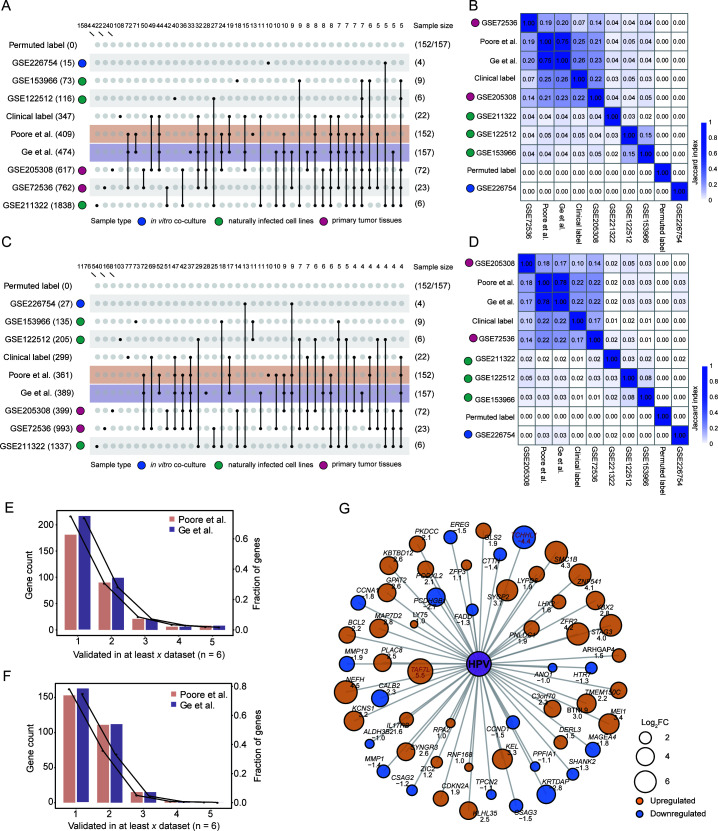
External data sets validate HPV-HNSC gene signatures in a model-dependent manner. (**A and B**) Validation of HPV-driven upregulated differentially expressed genes (DEGs) in HNSC. (**A**) Upset plot showing the intersections of upregulated gene sets from TCGA sources (including clinical and permuted labels as controls) and external validation cohorts. The number of genes in each specific intersection is shown at the top. External cohorts are colored by model type: *in vitro* co-cultures (blue), naturally infected cell lines (green), and primary tumor tissues (red). (**B**) Heatmap showing the pairwise JI quantifying the similarity between all upregulated gene sets. (**C and D**) Validation of HPV-driven downregulated DEGs in HNSC. (**C**) Upset plot, as in panel **A**, showing the intersections of downregulated gene sets. (**D**) Heatmap of pairwise JIs, as in panel **B**, for the downregulated gene sets. (**E and F**) Validation stringency plots for (**E**) upregulated and (**F**) downregulated genes from the TMPs of Sepich-Poore et al. (orange) and Ge et al. (purple). The plot shows the number of genes (bars, left *y*-axis) and the fraction of the total gene list (line, right *y*-axis) that were validated in at least *x* external data sets. (**G**) Network of robustly validated HPV-associated genes (validated in ≥3 external cohorts). Node size is proportional to the absolute log_2_ fold change (log_2_FC) from the TMP of Sepich-Poore et al., and color indicates the direction of regulation (orange: upregulated; blue: downregulated).

We first examined the well-established association between HPV and HNSC, comparing the gene signatures from both TMPs against six independent data sets (Fig. 4A through D). To establish an upper-bound benchmark, we also evaluated the signature derived from the TCGA’s own clinical HPV labels, which yielded high concordance with the external data sets (JIs up to 0.22). The TMP-derived signatures performed at a comparable level, with JIs reaching up to 0.23 for upregulated genes and 0.22 for downregulated genes. This strong concordance was also evident in the proportion of recovered genes; for example, 74.8% of upregulated genes from Sepich-Poore et al. and 73.6% from Ge et al. were validated in at least one external data set (Fig. 4E and F; Table S16). A more stringent criterion, requiring validation in at least two data sets, still confirmed an average of more than 30% of the identified genes. Among the most robustly validated hits, replicated in at least three cohorts, were the upregulated *TAF7L* (log₂FC = 5.5 and 5.9, adjusted *P* = 2.04 × 10^−5^ and 6.91 × 10^−6^ for Sepich-Poore et al. and Ge et al., respectively) and downregulated *TCHHL1* (log₂FC = −4.4 and −4.1, adjusted *P* = 1.86 × 10^−9^ and 4.74 × 10^−7^), both with established roles in the HPV life cycle (Fig. 4G) (32, 33).

A critical insight from this analysis was that validation success was highly model-dependent. We observed a clear hierarchy of concordance: it was strongest with data from external primary tumors (average JI = 0.101), followed by naturally infected cell lines (average JI = 0.023), and was lowest for *in vitro* co-culture models (average JI = 0.001). This quantitative trend suggests that the degree to which an experimental model recapitulates the native *in vivo* tumor microenvironment directly impacts its ability to validate computational predictions from patient samples.

In contrast to the strong validation for HPV in HNSC, we observed limited concordance for other microbe-host associations when compared against external data sets. This included HPV in CESC, EBV in STAD, *H. pylori* in STAD, HBV in LIHC, and *Fusobacterium nucleatum* in COAD (Tables S17 to S22). This lack of validation does not necessarily invalidate the original associations but rather highlights a critical challenge: the vast majority of available public data sets for these microbes are derived from *in vitro* co-culture models. As our HNSC analysis showed, these models exhibit the lowest concordance, suggesting that current experimental systems may be inadequate for validating the subtle transcriptional signals associated with the low-biomass microbial communities found in many tumor types.

Overall, our external validation demonstrates that TMPs can successfully identify robust and reproducible host-gene expression signatures, particularly for strong biological signals like HPV in HNSC. However, it also reveals that the successful validation of other associations is currently hampered by the limitations of available experimental data, emphasizing the need for more physiologically relevant validation systems.

### Survival associations revealed by lack of concordance and permutation benchmarking

Finally, we assessed the clinical relevance of the TMPs by evaluating associations between microbial presence and patient survival outcomes. Using a multivariable Cox proportional hazards regression, each TMP individually identified numerous microbes significantly associated with survival (adjusted *P* < 0.05; Fig. S11A and B; Table S23). The Sepich-Poore et al. profile yielded 12 overall survival (OS) and 5 progression-free survival (PFS) associations, while the Ge et al. profile produced 60 OS and 8 PFS associations. Critically, despite the large number of hits, there were only four overlaps between the prognostic microbes identified by the two independent TMPs.

To determine if these survival associations were statistically robust, we applied our permutation benchmarking framework. This analysis revealed that the number of survival-associated microbes found in the original data was not significantly greater than the number found in the randomly permuted data sets (permuted *P* > 0.05; Fig. S11C; Table S24). This indicates that the observed number of prognostic hits is consistent with what would be expected by random chance. Next, sensitivity analysis using 1,000 permutations revealed *P*-values and significance classifications highly consistent with those from the 100-permutation set (Fig. S10B through D). Furthermore, we found that 72.9% (62/85) significant associations from the original data were recurrently identified in the permuted data sets, demonstrating a profound lack of specificity and suggesting they are likely false positives (Fig. S11D; Table S24).

Taken together, the complete lack of concordance between the two TMPs and the failure to outperform a random null model in permutation testing provide strong evidence that these TMP-derived survival associations are statistically spurious. These findings highlight the profound challenge of identifying robust prognostic microbial biomarkers in TCGA data and underscore the need for extreme caution when interpreting such claims.

### MOMAC2: a stratified resource for exploring host-microbe interactions

**Fig 5:**
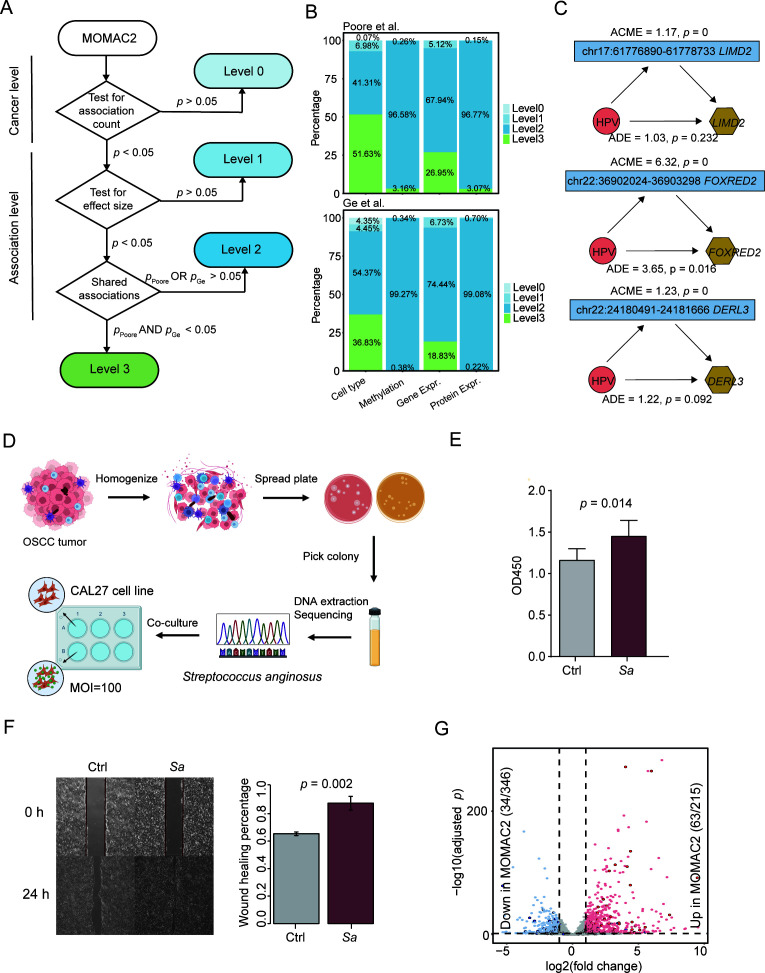
MOMAC2 as a stratified resource for discovering and validating host-microbe interactions. (**A**) Schematic of the four-level confidence stratification in MOMAC2, based on consistency and permutation testing. (**B**) Distribution of associations across the four confidence levels for each omics layer in the two TMPs. (**C**) Mediation analysis of three Level 3 HPV-gene associations in HNSC. The analysis confirms that the effect of HPV on gene expression is significantly mediated by CpG methylation (average causal mediation effect [ACME] *P* < 0.05). ACE, average causal effect. Red circle, microbe; blue box, promoter methylation; and yellow hexagon, gene expression. (**D**) Schematic of the experimental validation workflow for *Streptococcus anginosus*. (**E**) *S. anginosus* co-culture significantly promotes CAL27 cell proliferation after 72 h, as measured by a CCK-8 assay (*n* = 6 per group, two-tailed *t*-test). (**F**) *S. anginosus* co-culture significantly promotes CAL27 cell migration after 24 h in a wound healing assay (*n* = 3 per group, two-tailed *t*-test). (**G**) Concordance between differentially expressed genes identified in the *S. anginosus* co-culture and the high-confidence predictions from MOMAC2.

Our comprehensive benchmarking highlighted the variable reliability of host-microbe associations. To translate these findings into a practical resource for the research community, we developed MOMAC2, where all identified associations are stratified into four confidence levels based on their performance in our consistency and permutation tests (Fig. 5A; Table S25). The lowest-confidence tier (Level 0) contains associations from entire omics-cancer contexts (e.g., cell types in KIRC) where the total number of hits was not significant in permutation testing. Level 1 then flags individual associations whose effect size was not significantly greater than their permuted null distribution. The remaining, more statistically reliable associations are split into two tiers based on consistency. Level 2 represents associations with moderate confidence that are statistically robust in one TMP but not consistently detected across both TMPs. Finally, the highest-confidence tier, Level 3, is reserved for the most robust associations, which are those found in both TMPs and that pass permutation testing in both data sets. The majority of associations for methylation, gene, and protein expression were classified as moderate or high confidence (Level 2 or 3), providing a rich data set for exploration (Fig. 5B).

To operationalize these findings, we developed MOMAC2, an open-access, interactive web portal (https://comics.med.sustech.edu.cn/momac2) designed for the robust exploration of host-microbe interactions. The platform centers on a flexible search interface (Fig. S12) where users can query specific microbes, cell types, or molecular features. The output provides detailed statistics, crucially supplemented by our permutation-based reliability metrics and enrichment details. For researchers conducting systematic analyses, the portal also supports batch downloading of the full data set, enabling independent, customized filtering for data-driven hypothesis generation.

### MOMAC2 enables novel biological insights and experimental validation

To demonstrate the utility of MOMAC2 for generating and testing biological hypotheses, we first used it to explore putative regulatory mechanisms. We interrogated Level 2/3 associations where microbes were linked to both promoter CpG island methylation and the expression of the same gene—a known mechanism of microbial pathogenesis (34–37). We identified 192 such regulatory axes, primarily in UCEC, HNSC, and BRCA (Table S26). Of them, 70.3% (135/192) correlated with promoter hypermethylation and concomitant gene downregulation, while the remaining 29.7% were linked to promoter hypomethylation with concurrent gene upregulation. For example, mediation analysis of three Level 3 HPV-associated genes in HNSC (*LIMD2*, *FOXRED2*, and *DERL3*) confirmed that HPV’s effect on their expression is significantly mediated by promoter hypomethylation (*P* < 0.05; Fig. 5C; Table S27), a finding consistent with previous reports (38). Similarly, we identified a Level 2 association where EBV infection was linked to promoter hypermethylation and suppression of the *P2RY2* gene, recapitulating known EBV-driven epigenetic silencing (39).

Next, we used MOMAC2 to guide a novel experimental investigation. While *Streptococcus anginosus* has been implicated in gastric cancer (6, 40), MOMAC2 revealed numerous high-confidence gene expression associations for this bacterium in HNSC as well. This prompted us to investigate its functional role in oral cancer. We isolated a clinical strain of *S. anginosus* from human oral cancer tissue and co-cultured it with the CAL27 oral cancer cell line (Fig. 5D). Strikingly, co-culture significantly promoted CAL27 cell proliferation (*P* = 0.014, Fig. 5E) and migration in a wound-healing assay (*P* = 0.002, Fig. 5F). We then performed transcriptomic analysis and found strong validation for the database’s predictions, with 29.3% (63 of 215) of the predicted upregulated genes being recapitulated in our model (Fig. 5G; Table S28).

These results demonstrate the power of a rigorously stratified resource like MOMAC2 to move from large-scale computational screening to validated biological discovery, providing the community with a powerful tool to generate and prioritize testable hypotheses for future mechanistic studies.

## DISCUSSION

While the study of tumor microbiota using archives like TCGA offers a powerful lens into the cancer ecosystem, discrepancies in database selection, taxonomic classification tools, and decontamination strategies among representative TMPs have led to inconsistencies in microbial quantification. Consequently, these methodological variations result in contradictory associations with clinical features, questioning the reliability of such analyses (5, 41).

Our study contributes to this discussion by systematically comparing two state-of-the-art TCGA microbial profiles. We find that while the profiles show substantial agreement in microbial composition, their concordance in downstream host-microbe association analyses is moderate and highly dependent on the omics layer. Furthermore, our permutation-based benchmarking suggests that while many associations are statistically robust, others, particularly those involving cell type composition and patient survival, may be spurious within the current statistical framework. These observations underscore the critical importance of a multifaceted validation approach in cancer microbiome research.

Our findings help to quantitatively frame the ongoing controversy surrounding TMP reliability. The consistent detection of a core microbial signal across different pipelines, exemplified by the robust validation of HPV-driven gene expression in HNSC, speaks to the genuine potential of these resources. At the same time, the challenges we observed in accurately detecting other oncomicrobes like *H. pylori* and the striking discordance for methylation and protein-level associations serve as a practical demonstration of the field’s methodological sensitivities, especially in low-biomass settings.

In response to these challenges, we proposed and implemented a multi-tiered framework to help assess the reliability of host-microbe associations. Our permutation benchmarking offers a method to estimate the statistical robustness of findings against a null model, a step we found useful for flagging potentially spurious signals. We integrated these principles into MOMAC2, a publicly available web portal that stratifies associations by confidence level. This resource is intended to help researchers navigate the complexity of these data by providing layers of evidence, thereby facilitating more informed hypothesis generation.

The potential utility of such a structured approach can be seen in its application to biological discovery. Using high-confidence associations from MOMAC2, we identified putative methylation-mediated regulatory axes for known oncoviruses and, importantly, used the MOMAC2 to guide a novel experimental study. Our successful *in vitro* validation of *Streptococcus anginosus* in oral cancer cells serves as a practical example of how a carefully annotated computational resource can be used to generate and experimentally test new hypotheses. Moreover, while our study provides a systematic evaluation of host-microbe associations, we emphasize that the identified associations between specific microbes and host molecular signatures should be interpreted with caution. Specifically, such interpretations could be refined by considering the intracellular localization of microbes within particular cell types and the downstream impact of their metabolites on the tumor microenvironment. These correlations serve as computational hypotheses that require further validation through rigorous *in vivo* and *in vitro* experimental models.

Despite the insights gained, our study has several limitations that should be considered. First, our analytical framework did not explicitly account for potential batch effects due to the limited sample size within each cancer type, which may affect the reproducibility and generalizability of some results (42). Second, our evaluation was constrained to two representative TMPs; the inclusion of additional pipelines or independently processed data sets could offer a more integrative view of analytical consistency (21, 22). Third, while our *in vitro* co-culture models provided functional support for some transcriptomic associations, further *in vivo* validation and mechanistic investigations are necessary to establish causal relationships between microbial presence and oncogenic phenotypes. Finally, our analysis focused primarily on bacterial microbiota, and future studies incorporating viral and fungal communities would help elucidate a more comprehensive ecological portrait of the tumor microenvironment.

Collectively, our findings advocate for a nuanced “trust but verify” approach to tumor microbiome analysis. The debate in the field can fruitfully move beyond questions of simple presence or absence toward establishing standards for robust, reproducible functional analysis. We believe that combining cross-pipeline validation with rigorous statistical benchmarking, as explored in this study, represents a promising direction. Continued efforts to integrate improved computational frameworks with physiologically relevant experimental models will be essential to fully elucidate the clinical significance of the tumor microbiome.

## MATERIALS AND METHODS

### TCGA-derived microbial profile data collection and processing

We obtained two recently updated tumor microbial profiles derived from TCGA data, published by Sepich-Poore et al. (5) (*n* = 7,119) and Ge et al. (17) (*n* = 5,734). To ensure data quality, we focused on primary tumor samples (*n* = 2,060 and 2,644, respectively) with TMPs derived from WGS, yielding a non-redundant sample set of 1,835 tumors from Sepich-Poore et al. and 2,300 from Ge et al., spanning 24 cancer types. Among these, 1,808 samples were shared between the two data sets (Fig. S1A; Table S1).

Sepich-Poore et al. initially depleted host sequences by aligning reads against multiple versions of the human references (hg19, hg38, and T2T-CHM13). The remaining sequences were then mapped to the RS210-clean database, which had been pre-scrubbed. Woltka (43) was employed to assign taxonomic classifications, and the final TMPs were generated by retaining microbes consistent across multiple cohorts and those with reads uniformly distributed across their respective microbial genomes. Hence, for Sepich-Poore et al., we utilized the RS210-clean filtered microbial read count data, which included 583 species (325 bacteria, 255 viruses, and 3 fungi; Fig. S1B). Raw read counts were normalized to counts per million reads sequenced (CPM) using the total read count per sample, and values less than 0.01 were labeled as zero, consistent with Ge et al. Similarly, Ge et al. depleted host sequences by aligning reads against three versions of the human reference genomes, followed by mapping to the Microbial2023 and Fungi_RefSeq databases using KrakenUniq. Although potential contaminating species were extensively discussed in their study, a total of 11,895 microbial species (comprising 11,338 prokaryotes and 557 fungi) remained available for analysis. Consequently, we collected this data set as one of our primary TMPs.

To focus on the core microbiota while minimizing sequencing noise and potential false positives, we employed a dual-filtering strategy: retaining species present in both data sets and those with a relative abundance exceeding 1% in at least one cancer type (Fig. S1C). This approach yielded 268 species for the Sepich-Poore et al. data set (256 bacteria, 9 viruses, and 3 fungi) and 352 for Ge et al. (299 bacteria, 14 viruses, and 39 fungi), with a high degree of inter-study consistency represented by 231 shared taxa (accounting for 86.19% and 65.62% of each data set, respectively). Sensitivity analyses revealed that although relaxing the threshold to 0.1% or 0.01% increased the taxon count by 3–10-fold, the proportion of shared taxa declined to 68.89% and 55.14% in Sepich-Poore et al. and to 30.43% and 11.71% in Ge et al., respectively.

### TCGA multi-omics data collection and processing

For host molecular features, the following data sets were obtained from the UCSC Xena-TCGA Pan-Cancer server: DNA methylation data (Illumina 450K array, *n* = 9,639), RNA expression data (batch-effect normalized mRNA data, *n* = 11,060), and protein expression data (*n* = 7,744) (44). For DNA methylation analysis, β-values of CpG sites were first converted to *M*-values, and mean *M*-values were calculated as methylation levels of CpG islands via the minfi package (version 1.52.1) (45). For host gene expression data, we utilized the biomaRt package (46) to retain only protein-coding genes with the top 75% variance across all genes. Gene names, CpG islands, and proteins were mapped using TCGA annotations and the official manifest file for Illumina Infinium HumanMethylation450 v1.2 BeadChip. Additionally, we also collected the abundance of 20 different cell types for 10,490 TCGA tumors, estimated by deconvolution of bulk RNA-Seq data using Kassandra (47). In total, our study incorporated multi-omics data covering 20 cell types, 10,869 genes, 25,493 CpG islands, and 218 proteins, all of which were subsequently correlated with TMPs.

### Consistency analysis of TMPs

The consistency between the two TMPs was systematically evaluated through a multifaceted analysis. First, agreement for each of the 231 microbes in the presence/absence calls was evaluated using Fisher’s exact test. Second, the concordance of abundance patterns (measured in CPM) across the 1,808 matched samples was assessed using Spearman’s rank correlation. Alpha diversity indices (Shannon and Simpson indices) were calculated using the vegan package (v2.7.1). Finally, the overall compositional concordance of the TMPs, both within and between cancer types, was quantified by Jaccard distance and Jensen-Shannon Divergence. These metrics were implemented using the vegan (v2.7.1) and philentropy (v0.9.0) R packages, respectively. For all tests, *P*-values were adjusted for multiple comparisons using the BH method, and an adjusted *P*-value < 0.05 was considered statistically significant.

### Benchmarking of oncomicrobe detection

The clinical detection status of oncogenic microbes was obtained from the “clinical_PANCAN_patient_with_followup.tsv” file downloaded from NCI Genomic Data Commons (https://gdc.cancer.gov/node/905/). Specific definitions were as follows: in HNSC, HPV status was determined by two methods: *in situ* hybridization (“by_ish_testing”) identified 7 positives and 17 negatives, and p16INK4A immunohistochemistry (“by_p16_testing”) identified 9 positives and 17 negatives. For CESC, HPV status with specific subtypes was provided. Since the tumor microbiome profiles detected Alphapapillomavirus_9 (a species that includes HPV16 but not HPV18), HPV16 was considered positive, resulting in seven positive and one negative case. *H. pylori* infection status was directly based on the “h_pylori_infection” clinical label, comprising 3 positive and 19 negative cases. HBV infection was determined from the “hbv_test” label, where a positive “hepatitis B surface antigen” result defined HBV positivity, including 24 positive and 1 negative case. EBV status was not based on clinical labels but rather on independent prior studies that reported EBV viral loads (cpm) (31), encompassing 43 positive and 133 negative samples.

Recall, precision, and F1 score were calculated to evaluate the performance of TMPs in detecting the clinical pathogen status. Recall was calculated as true positive/(true positive + false negative). Precision was calculated as true positive/(true positive + false positive). F1 score was calculated as the harmonic mean of precision and recall: 2 × (precision × recall)/(precision + recall).

For each microbe-pathogen pair, a 2 × 2 contingency table was constructed based on the presence/absence calls in the TMP and the positive/negative clinical label. The test was performed using the fisher.test function in R, which computes the exact *P*-value assessing the null hypothesis of independence between the two binary variables.

### Host-microbe association analysis

In the association analysis, for each cancer type, we divided the samples into two groups based on the presence or absence of each microbe. We then used the two-tailed Wilcoxon test to test the differences between the two groups when the usable number of samples was ≥3 in each group. The *P*-values were corrected for multiple testing using the Benjamini-Hochberg method within each combination of cancer type and host feature type. Associations with an adjusted *P*-value < 0.1 were considered significant. Additionally, for epigenome and transcriptome, an absolute fold change >2 was considered significant. To quantify the similarity of significant association sets (e.g., between different data sets or microbial strains), the JI was calculated as the size of the intersection divided by the size of the union of the two sets.

### Permutation-based benchmarking

To generate null distributions, the sample labels within each TMP were randomly permuted 100 times independently. During each permutation iteration, the presence/absence ratio of each microbe was kept unchanged to maintain the original sparsity structure of the data. Subsequently, host-microbe associations were identified in the permuted TMP data sets using identical statistical methods and criteria as those applied to the original TMPs. Given the potential unreliability of cell-type and survival associations, the number of permutations was further increased to 1,000 to demonstrate the robustness of the findings.

For each cancer type, the total number of significant associations observed in the original data was compared against the null distribution of association counts generated from the 100 permuted data sets. The permuted *P*-value was calculated as the proportion of permutations in which the number of significant associations was equal to or greater than the observed count.

For each specific host-microbe association identified in the original analysis, its fold change was compared to the null distribution of fold change for that specific pair derived from the permutations. The permuted *P*-value was calculated to assess the probability of observing an effect size of that magnitude by chance alone. This *P*-value reflects the reliability of each individual association.

To assess the consistency of associations between the two original TMPs, the JI was calculated for the sets of significant associations. The significance of this observed JI was evaluated by comparing it against a null distribution of JI values. This null distribution was generated by calculating the JI between sets of significant associations derived from randomly permuted data sets within the same cancer type. A *P*-value was computed to determine if the overlap between the two real TMPs was significantly greater than expected by chance.

### Validation gene expression data collection and processing

To validate host-microbe associations, we curated 26 tumor gene expression data sets from the Gene Expression Omnibus in total (see Table S15 for accession numbers). These data sets were categorized into three main types: (i) gene expression data from cancer cell lines co-cultured with cancer-associated microbes, including HPV, HBV, EBV, and *H. pylori* (*n* = 16); (ii) gene expression data comparing cancer cell lines with or without natural HPV or EBV infection (*n* = 5); and (iii) gene expression data from tumor tissues stratified by HPV, EBV, or *H. pylori* infection status (*n* = 5).

Differential gene expression analysis was performed using DESeq2 (48), focusing on protein-coding genes. Multiple testing correction was applied using the BH method, and genes with an adjusted *P*-value <0.05 and an absolute fold change >2 were considered significantly differentially expressed. The lists of upregulated and downregulated genes were separately compared against those identified in MOMAC2, and the degree of overlap was quantified using the JI.

### Survival analysis

Curated survival phenotype data, including overall survival and progression-free survival, were obtained from the UCSC Xena server (44). For each cancer type, samples were stratified into presence and absence groups. The prognostic impact of microbial abundance on OS and PFS was assessed using multivariable Cox proportional hazards regression. We integrated the following confounding factors into the models (Table S1): age, gender (male vs female), race (White vs non-White), AJCC pathologic tumor stage (early [stages I–II] vs advanced [stages III–IV]), and treatment status. Patients were categorized as “Yes” for treatment if they had a record of any of the following: radiation therapy, postoperative prescription (Rx) and treatment (Tx), targeted molecular therapy, additional pharmaceutical therapy, or additional radiation therapy. Other covariates included tumor purity and sequencing center (including Baylor College of Medicine, Broad Institute, Harvard Medical School, MD Anderson, and Washington University). Multiple testing correction was applied using the BH method, and associations with an adjusted *P*-value < 0.05 were considered statistically significant.

We used the same permuted null distribution as generated for the omics association analysis to benchmark the survival associations. This approach provided a two-tiered reliability assessment: evaluation of cancer-type-wide credibility by comparing significant OS/PFS associations count to a null model, and evaluation of individual associations through empirical *P*-values.

### MOMAC2 stratification

To provide a robustly annotated resource, we introduced a four-level confidence classification for all associations in MOMAC2, based on permutation testing: Level 0, associations derived from cancer types where the overall number of significant hits was not greater than the random expectation (permutation *P* > 0.05); Level 1, individual associations whose fold change was not significantly greater than the null distribution generated by permutation testing; Level 2, associations show significance but are either driven by non-shared microbes or lack simultaneous significance across both TMPs; Level 3, associations involving a microbe shared across TMPs that achieved statistical significance in both data sets.

### Mediation analysis

To investigate methylation-mediated regulation of gene expression by microbes, a tripartite mediation analysis was performed. This analysis tested whether the effect of HPV status on gene expression (Table S26) was mediated by CpG island methylation. For each triplet, two linear models were fit using the mediation (v4.5.1) package: (i) a model regressing the methylation mediator (*M*) on the microbial independent variable (*X*) and (ii) a model regressing the gene expression outcome (*Y*) on both the microbial variable (*X*) and the methylation mediator (*M*). The significance of the mediation effect, represented by the average causal mediation effect (ACME), was assessed using a non-parametric bootstrap approach with 5,000 simulations. The average direct effect (ADE) and the proportion mediated were also calculated. To account for multiple testing, *P*-values for the ACME and ADE were adjusted using the BH method. A mediation effect was considered statistically significant if the adjusted ACME *P*-value was less than 0.05.

### Isolation and culture of intratumoral microbes

Two fresh oral cancer tissue samples from the Shenzhen People’s Hospital were collected and transported in MACS Tissue Storage Solution (Miltenyi) at 4°C within 2 h. Tissues were homogenized in 1× PBS and plated on Brain Heart Infusion agar for microbial isolation. Plates were incubated under both aerobic and anaerobic conditions at 37°C for up to 3 days. Single colonies with distinct morphologies were subcultured to obtain pure isolates.

### Microbial genomic DNA extraction, sequencing, and identification

Genomic DNA was extracted from pure bacterial cultures using the FastPure Bacteria DNA Isolation Mini Kit (Vazyme). Whole-genome sequencing was performed on the DNBSEQ-T7 platform, generating 150 bp paired-end reads with an average sequencing depth of 1 Gb. Adapters and low-quality reads were trimmed using Trimmomatic (v0.39) (49). Cleaned reads were assembled into contigs using Megahit (v1.2.9) (50), and contigs with a length greater than 500 bp were retained for subsequent analysis. The assembled genomes were used for species identification by comparing against the GTDB reference database R207 using GTDB-Tk (v2.1.1) (51).

### Co-culture assays

We co-cultured *S. anginosus* with CAL27 oral cancer cells on a 6-well and 96-well plate at a multiplicity of infection of 100. CAL27 cells were seeded in 6-well plates at a density of 50,000 cells per well or in 96-well plates at 2,000 cells per well. After 2 h of treatment, the cells were washed three times with PBS, followed by incubation in medium containing 200 µM gentamicin for 24 h (6 well) or 72 h (96 well). Cells from the 6-well plates were used for RNA extraction and the wound healing assay, while those from the 96-well plates were subjected to cell viability analysis using the CCK-8 assay. The CCK-8 reagent was added to the 96-well plate, and the absorbance was measured after a 2-h incubation.

### RNA sequencing and differential gene expression analysis

Total RNA was extracted from *S. anginosus*-treated CAL27 cells (*n* = 3) and control (*n* = 3) using the Cell Total RNA Kit (Yeasen, China). RNA-seq was performed by Genergy Biotechnology, then the sequencing reads were processed by mapping on the hg38 reference by STAR (v2.7.11a) (52) with the default options. Differential gene expression was calculated using DESeq2 (v1.49.4) (48).

## Data Availability

The summary statistics of association tests have been uploaded to the open-access MOMAC2 web portal (https://comics.med.sustech.edu.cn/momac2) and are available for download to all users. All code and supplementary data are available at https://github.com/comics-bio/Analysis_for_MOMAC_v2. The raw sequencing data have been deposited in the National Genomics Data Center (NGDC) under BioProject number PRJCA060385 with accession number CRA040193 for WGS and HRA017378 for RNA-Seq.

## SUPPLEMENTAL MATERIAL

Supplemental material msystems.00180-26-s0001.pdf Figures S1-S12 and supplemental table captions.

Supplemental tables part 1 msystems.00180-26-s0002.xlsx Tables S1-S15.

Supplemental tables part 2. msystems.00180-26-s0003.xlsx Tables S16-S28.

ASM does not own the copyrights to Supplemental Material that may be linked to, or accessed through, an article. The authors have granted ASM a non-exclusive, world-wide license to publish the Supplemental Material files. Please contact the corresponding author directly for reuse.

## References

[1] Battaglia TW, Mimpen IL, Traets JJH, van Hoeck A, Zeverijn LJ, Geurts BS, de Wit GF, Noë M, Hofland I, Vos JL, Cornelissen S, Alkemade M, Broeks A, Zuur CL, Cuppen E, Wessels L, van de Haar J, Voest E. 2024. A pan-cancer analysis of the microbiome in metastatic cancer. Cell 187:2324–2335. doi:10.1016/j.cell.2024.03.021 38599211

[2] Dohlman AB, Klug J, Mesko M, Gao IH, Lipkin SM, Shen X, Iliev ID. 2022. A pan-cancer mycobiome analysis reveals fungal involvement in gastrointestinal and lung tumors. Cell 185:3807–3822. doi:10.1016/j.cell.2022.09.015 36179671 PMC9564002

[3] Narunsky-Haziza L, Sepich-Poore GD, Livyatan I, Asraf O, Martino C, Nejman D, Gavert N, Stajich JE, Amit G, González A, et al.. 2022. Pan-cancer analyses reveal cancer-type-specific fungal ecologies and bacteriome interactions. Cell 185:3789–3806. doi:10.1016/j.cell.2022.09.005 36179670 PMC9567272

[4] Nejman D, Livyatan I, Fuks G, Gavert N, Zwang Y, Geller LT, Rotter-Maskowitz A, Weiser R, Mallel G, Gigi E, et al.. 2020. The human tumor microbiome is composed of tumor type-specific intracellular bacteria. Science 368:973–980. doi:10.1126/science.aay9189 32467386 PMC7757858

[5] Sepich-Poore GD, McDonald D, Kopylova E, Guccione C, Zhu Q, Austin G, Carpenter C, Fraraccio S, Wandro S, Kosciolek T, Janssen S, Metcalf JL, Song SJ, Kanbar J, Miller-Montgomery S, Heaton R, Mckay R, Patel SP, Swafford AD, Korem T, Knight R. 2024. Robustness of cancer microbiome signals over a broad range of methodological variation. Oncogene 43:1127–1148. doi:10.1038/s41388-024-02974-w 38396294 PMC10997506

[6] Fu K, Cheung AHK, Wong CC, Liu W, Zhou Y, Wang F, Huang P, Yuan K, Coker OO, Pan Y, Chen D, Lam NM, Gao M, Zhang X, Huang H, To KF, Sung JJY, Yu J. 2024. Streptococcus anginosus promotes gastric inflammation, atrophy, and tumorigenesis in mice. Cell 187:882–896. doi:10.1016/j.cell.2024.01.004 38295787

[7] Liu B, Zhou Z, Jin Y, Lu J, Feng D, Peng R, Sun H, Mu X, Li C, Chen Y. 2022. Hepatic stellate cell activation and senescence induced by intrahepatic microbiota disturbances drive progression of liver cirrhosis toward hepatocellular carcinoma. J Immunother Cancer 10:e003069. doi:10.1136/jitc-2021-003069 34996812 PMC8744134

[8] Fu A, Yao B, Dong T, Chen Y, Yao J, Liu Y, Li H, Bai H, Liu X, Zhang Y, Wang C, Guo Y, Li N, Cai S. 2022. Tumor-resident intracellular microbiota promotes metastatic colonization in breast cancer. Cell 185:1356–1372. doi:10.1016/j.cell.2022.02.027 35395179

[9] Guo S, Chen J, Chen F, Zeng Q, Liu W-L, Zhang G. 2020. Exosomes derived from Fusobacterium nucleatum-infected colorectal cancer cells facilitate tumour metastasis by selectively carrying miR-1246/92b-3p/27a-3p and CXCL16. Gut 70:1507–1519. doi:10.1136/gutjnl-2020-321187 33172926

[10] Geller LT, Barzily-Rokni M, Danino T, Jonas OH, Shental N, Nejman D, Gavert N, Zwang Y, Cooper ZA, Shee K, et al.. 2017. Potential role of intratumor bacteria in mediating tumor resistance to the chemotherapeutic drug gemcitabine. Science 357:1156–1160. doi:10.1126/science.aah5043 28912244 PMC5727343

[11] Wang M, Rousseau B, Qiu K, Huang G, Zhang Y, Su H, Le Bihan-Benjamin C, Khati I, Artz O, Foote MB, et al.. 2024. Killing tumor-associated bacteria with a liposomal antibiotic generates neoantigens that induce anti-tumor immune responses. Nat Biotechnol 42:1263–1274. doi:10.1038/s41587-023-01957-8 37749267 PMC12892252

[12] Guillot N, Roméo B, Manesh SS, Milano G, Brest P, Zitvogel L, Hofman P, Mograbi B. 2023. Manipulating the gut and tumor microbiota for immune checkpoint inhibitor therapy: from dream to reality. Trends Mol Med 29:897–911. doi:10.1016/j.molmed.2023.08.004 37704493

[13] Sun L, Ke X, Guan A, Jin B, Qu J, Wang Y, Xu X, Li C, Sun H, Xu H, Xu G, Sang X, Feng Y, Sun Y, Yang H, Mao Y. 2023. Intratumoural microbiome can predict the prognosis of hepatocellular carcinoma after surgery. Clin Transl Med 13:e1331. doi:10.1002/ctm2.1331 37462602 PMC10353526

[14] White MG, Damania A, Alshenaifi J, Sahasrabhojane P, Peacock O, Losh J, Wong MC, Lutter-Berkova Z, Chang GJ, Futreal A, Wargo JA, Ajami NJ, Kopetz S, You YN. 2023. Young-onset rectal cancer: unique tumoral microbiome and correlation with response to neoadjuvant therapy. Ann Surg 278:538–548. doi:10.1097/SLA.0000000000006015 37465976 PMC10528779

[15] Wu H, Leng X, Liu Q, Mao T, Jiang T, Liu Y, Li F, Cao C, Fan J, Chen L, et al.. 2023. Intratumoral microbiota composition regulates chemoimmunotherapy response in esophageal squamous cell carcinoma. Cancer Res 83:3131–3144. doi:10.1158/0008-5472.CAN-22-2593 37433041

[16] Ghaddar B, Biswas A, Harris C, Omary MB, Carpizo DR, Blaser MJ, De S. 2022. Tumor microbiome links cellular programs and immunity in pancreatic cancer. Cancer Cell 40:1240–1253. doi:10.1016/j.ccell.2022.09.009 36220074 PMC9556978

[17] Ge Y, Lu J, Puiu D, Revsine M, Salzberg SL. 2024. Comprehensive analysis of microbial content in whole-genome sequencing samples from The Cancer Genome Atlas project. bioRxiv:2024.05.24.595788. doi:10.1101/2024.05.24.595788 PMC12821378 40901923

[18] Fletcher AA, Kelly MS, Eckhoff AM, Allen PJ. 2023. Revisiting the intrinsic mycobiome in pancreatic cancer. Nature 620:E1–E6. doi:10.1038/s41586-023-06292-1 37532819 PMC11062486

[19] Gihawi A, Cooper CS, Brewer DS. 2023. Caution regarding the specificities of pan-cancer microbial structure. Microb Genom 9:mgen001088. doi:10.1099/mgen.0.001088 37555750 PMC10483429

[20] Gihawi A, Ge Y, Lu J, Puiu D, Xu A, Cooper CS, Brewer DS, Pertea M, Salzberg SL. 2023. Major data analysis errors invalidate cancer microbiome findings. mBio 14:e01607-23. doi:10.1128/mbio.01607-23 37811944 PMC10653788

[21] Chen KP, Hsu CL, Oyang YJ, Huang HC, Juan HF. 2023. BIC: a database for the transcriptional landscape of bacteria in cancer. Nucleic Acids Res 51:D1205–D1211. doi:10.1093/nar/gkac891 36263784 PMC9825443

[22] Rodriguez RM, Hernandez BY, Menor M, Deng Y, Khadka VS. 2020. The landscape of bacterial presence in tumor and adjacent normal tissue across 9 major cancer types using TCGA exome sequencing. Comput Struct Biotechnol J 18:631–641. doi:10.1016/j.csbj.2020.03.003 32257046 PMC7109368

[23] Sheng D, Jin C, Yue K, Yue M, Liang Y, Xue X, Li P, Zhao G, Zhang L. 2024. Pan-cancer atlas of tumor-resident microbiome, immunity and prognosis. Cancer Lett 598:217077. doi:10.1016/j.canlet.2024.217077 38908541

[24] Dohlman AB, Arguijo Mendoza D, Ding S, Gao M, Dressman H, Iliev ID, Lipkin SM, Shen X. 2021. The cancer microbiome atlas: a pan-cancer comparative analysis to distinguish tissue-resident microbiota from contaminants. Cell Host Microbe 29:281–298. doi:10.1016/j.chom.2020.12.001 33382980 PMC7878430

[25] Schorr L, Mathies M, Elinav E, Puschhof J. 2023. Intracellular bacteria in cancer-prospects and debates. NPJ Biofilms Microbiomes 9:76. doi:10.1038/s41522-023-00446-9 37813921 PMC10562400

[26] Zhang H, Xiong X, Cheng M, Ji L, Ning K. 2024. Deep learning enabled integration of tumor microenvironment microbial profiles and host gene expressions for interpretable survival subtyping in diverse types of cancers. mSystems 9:e01395-24. doi:10.1128/msystems.01395-24 39565103 PMC11651096

[27] Li X, Wu D, Li Q, Gu J, Gao W, Zhu X, Yin W, Zhu R, Zhu L, Jiao N. 2024. Host-microbiota interactions contributing to the heterogeneous tumor microenvironment in colorectal cancer. Physiol Genomics 56:221–234. doi:10.1152/physiolgenomics.00103.2023 38073489

[28] Liu Z, Sun Y, Li Y, Ma A, Willaims NF, Jahanbahkshi S, Hoyd R, Wang X, Zhang S, Zhu J, Xu D, Spakowicz D, Ma Q, Liu B. 2024. An explainable graph neural framework to identify cancer-associated intratumoral microbial communities. Adv Sci (Weinheim) 11. doi:10.1002/advs.202403393 PMC11538693 39225619

[29] Gao Y, Zhang H, Chu D, Ning K. 2025. Intra-tumor microbiome-based tumor survival indices predict immune interaction and drug sensitivity on pan-cancer scale. mSystems 10:e00312-25. doi:10.1128/msystems.00312-25 40558028 PMC12282056

[30] Hermida LC, Gertz EM, Ruppin E. 2022. Predicting cancer prognosis and drug response from the tumor microbiome. Nat Commun 13:2896. doi:10.1038/s41467-022-30512-3 35610202 PMC9130323

[31] Rooney MS, Shukla SA, Wu CJ, Getz G, Hacohen N. 2015. Molecular and genetic properties of tumors associated with local immune cytolytic activity. Cell 160:48–61. doi:10.1016/j.cell.2014.12.033 25594174 PMC4856474

[32] Slebos RJC, Yi Y, Ely K, Carter J, Evjen A, Zhang X, Shyr Y, Murphy BM, Cmelak AJ, Burkey BB, Netterville JL, Levy S, Yarbrough WG, Chung CH. 2006. Gene expression differences associated with human papillomavirus status in head and neck squamous cell carcinoma. Clin Cancer Res 12:701–709. doi:10.1158/1078-0432.CCR-05-2017 16467079

[33] Gunasekharan VK, Li Y, Andrade J, Laimins LA. 2016. Post-transcriptional regulation of KLF4 by high-risk human papillomaviruses is necessary for the differentiation-dependent viral life cycle. PLoS Pathog 12:e1005747. doi:10.1371/journal.ppat.1005747 27386862 PMC4936677

[34] Bird A. 2002. DNA methylation patterns and epigenetic memory. Genes Dev 16:6–21. doi:10.1101/gad.947102 11782440

[35] Herman JG, Baylin SB. 2003. Gene silencing in cancer in association with promoter hypermethylation. N Engl J Med 349:2042–2054. doi:10.1056/NEJMra023075 14627790

[36] Kuss-Duerkop SK, Westrich JA, Pyeon D. 2018. DNA tumor virus regulation of host DNA methylation and its implications for immune evasion and oncogenesis. Viruses 10:82. doi:10.3390/v10020082 29438328 PMC5850389

[37] Paschos K, Allday MJ. 2010. Epigenetic reprogramming of host genes in viral and microbial pathogenesis. Trends Microbiol 18:439–447. doi:10.1016/j.tim.2010.07.003 20724161 PMC3089700

[38] Degli Esposti D, Sklias A, Lima SC, Beghelli-de la Forest Divonne S, Cahais V, Fernandez-Jimenez N, Cros M-P, Ecsedi S, Cuenin C, Bouaoun L, Byrnes G, Accardi R, Sudaka A, Giordanengo V, Hernandez-Vargas H, Pinto LFR, Van Obberghen-Schilling E, Herceg Z. 2017. Unique DNA methylation signature in HPV-positive head and neck squamous cell carcinomas. Genome Med 9:33. doi:10.1186/s13073-017-0419-z 28381277 PMC5382363

[39] Zhao J, Liang Q, Cheung K-F, Kang W, Lung RWM, Tong JHM, To KF, Sung JJY, Yu J. 2013. Genome-wide identification of Epstein-Barr virus–driven promoter methylation profiles of human genes in gastric cancer cells. Cancer 119:304–312. doi:10.1002/cncr.27724 22833454

[40] Yuan L, Pan L, Wang Y, Zhao J, Fang L, Zhou Y, Xia R, Ma Y, Jiang Z, Xu Z, Hu C, Wang Y, Zhang S, Zhang B, Ding H, Chen M, Cheng H, Goel A, Zhang Z, Cheng X. 2024. Characterization of the landscape of the intratumoral microbiota reveals that Streptococcus anginosus increases the risk of gastric cancer initiation and progression. Cell Discov 10:117. doi:10.1038/s41421-024-00746-0 39587089 PMC11589709

[41] Wang Q, Liu Z, Ma A, Li Z, Liu B, Ma Q. 2023. Computational methods and challenges in analyzing intratumoral microbiome data. Trends Microbiol 31:707–722. doi:10.1016/j.tim.2023.01.011 36841736 PMC10272078

[42] Luo M, Liu Y, Hermida LC, Gertz EM, Zhang Z, Li Q, Diao L, Ruppin E, Han L. 2022. Race is a key determinant of the human intratumor microbiome. Cancer Cell 40:901–902. doi:10.1016/j.ccell.2022.08.007 36099885 PMC9887946

[43] Zhu Q, Huang S, Gonzalez A, McGrath I, McDonald D, Haiminen N, Armstrong G, Vázquez-Baeza Y, Yu J, Kuczynski J, et al.. 2022. Phylogeny-aware analysis of metagenome community ecology based on matched reference genomes while bypassing taxonomy. mSystems 7:e00167-22. doi:10.1128/msystems.00167-22 35369727 PMC9040630

[44] Goldman MJ, Craft B, Hastie M, Repečka K, McDade F, Kamath A, Banerjee A, Luo Y, Rogers D, Brooks AN, Zhu J, Haussler D. 2020. Visualizing and interpreting cancer genomics data via the Xena platform. Nat Biotechnol 38:675–678. doi:10.1038/s41587-020-0546-8 32444850 PMC7386072

[45] Aryee MJ, Jaffe AE, Corrada-Bravo H, Ladd-Acosta C, Feinberg AP, Hansen KD, Irizarry RA. 2014. Minfi: a flexible and comprehensive Bioconductor package for the analysis of Infinium DNA methylation microarrays. Bioinformatics 30:1363–1369. doi:10.1093/bioinformatics/btu049 24478339 PMC4016708

[46] Durinck S, Spellman PT, Birney E, Huber W. 2009. Mapping identifiers for the integration of genomic datasets with the R/Bioconductor package BiomaRt. Nat Protoc 4:1184–1191. doi:10.1038/nprot.2009.97 19617889 PMC3159387

[47] Zaitsev A, Chelushkin M, Dyikanov D, Cheremushkin I, Shpak B, Nomie K, Zyrin V, Nuzhdina E, Lozinsky Y, Zotova A, et al.. 2022. Precise reconstruction of the TME using bulk RNA-seq and a machine learning algorithm trained on artificial transcriptomes. Cancer Cell 40:879–894. doi:10.1016/j.ccell.2022.07.006 35944503

[48] Love MI, Huber W, Anders S. 2014. Moderated estimation of fold change and dispersion for RNA-seq data with DESeq2. Genome Biol 15:550. doi:10.1186/s13059-014-0550-8 25516281 PMC4302049

[49] Bolger AM, Lohse M, Usadel B. 2014. Trimmomatic: a flexible trimmer for Illumina sequence data. Bioinformatics 30:2114–2120. doi:10.1093/bioinformatics/btu170 24695404 PMC4103590

[50] Li D, Liu CM, Luo R, Sadakane K, Lam TW. 2015. MEGAHIT: an ultra-fast single-node solution for large and complex metagenomics assembly via succinct de Bruijn graph. Bioinformatics 31:1674–1676. doi:10.1093/bioinformatics/btv033 25609793

[51] Chaumeil PA, Mussig AJ, Hugenholtz P, Parks DH. 2022. GTDB-Tk v2: memory friendly classification with the genome taxonomy database. Bioinformatics 38:5315–5316. doi:10.1093/bioinformatics/btac672 36218463 PMC9710552

[52] Dobin A, Davis CA, Schlesinger F, Drenkow J, Zaleski C, Jha S, Batut P, Chaisson M, Gingeras TR. 2013. STAR: ultrafast universal RNA-seq aligner. Bioinformatics 29:15–21. doi:10.1093/bioinformatics/bts635 23104886 PMC3530905

