# Supplementary material for: Systematic evaluation of TCGA tumor microbiota reveals context-dependent reliability
Source: mSystems. 2026 Apr 22;11(5):e00180-26. doi: 10.1128/msystems.00180-26 (PMC13185545; doi:10.1128/msystems.00180-26)
Supplement: Supplemental material — Figures S1-S12 and supplemental table captions. [file msystems.00180-26-s0001.pdf]

Supplementary Information

Systematic Evaluation of TCGA Tumor Microbiota Reveals Context-Dependent Reliability

Chenchen Ma[1,2], Changxing Su[1,2], Jiaxuan Li[1,2], Jiaying Wang[1,2], Jianliang Liao[1,2], Lanlan Cheng[1,2], Jiuxin Qu[3], Guoquan Zhang[4], Jun Jiang[5]*, Shimin Shuai[1,2]*

1. Department of Human Cell Biology and Genetics, School of Medicine, Southern University of Science and Technology, Shenzhen 518055, Guangdong, China
2. SUSTech Homeostatic Medicine Institute, School of Medicine, Southern University of Science and Technology, Shenzhen 518055, Guangdong, China
3. Department of Clinical Laboratory, Shenzhen Third People's Hospital, National Clinical Research Center for Infectious Diseases, The Second Affiliated Hospital of Southern University of Science and Technology, Shenzhen 518114, Guangdong, China
4. Medical Center of Stomatology, Shenzhen People's Hospital (The First Affiliated Hospital, Southern University of Science and Technology; The Second Clinical Medical College, Jinan University), Shenzhen 518020, Guangdong, China
5. Department of Emergency Medicine, The First People's Hospital of Foshan (Foshan Hospital Affiliated to Southern University of Science and Technology), School of Medicine, Southern University of Science and Technology, Foshan 528000, Guangdong, China

* Correspondence: Jun Jiang (jiangjungd@163.com); Shimin Shuai (shuaism@sustech.edu.cn, lead contact)

Table of Content

Figure S1-S12

Titles for Table S1-S28

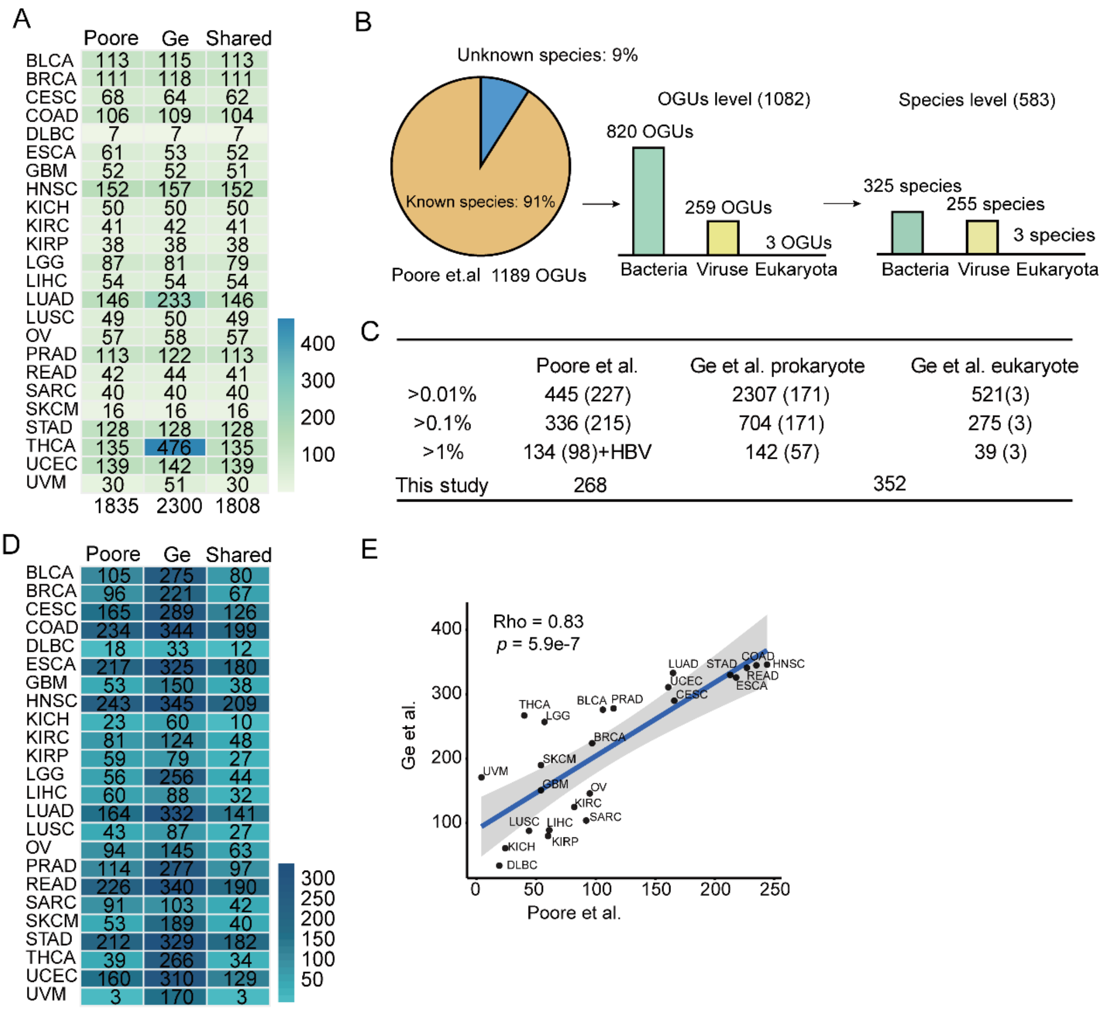

Figure S1. Sample and microbial size of the study. (A) Number of available and shared samples across 24 cancer types. (B) Richness of Operational Genomic Unit (OGU) and species in the TMP from Poore et al. Poore et al. identified a total of 1,189 OGUs, of which 1,082 (91%) were taxonomically assigned at the species level. These comprised 820 bacterial, 259 viral, and 3 eukaryotic OGUs, which collectively resolved into 583 distinct species (325 bacterial, 255 viral, and 3 eukaryotic). (C) Count of species with abundance exceeding thresholds of 0.01%, 0.1%, and 1% in any cancer type. A total of 268 species from Poore et al. and 352 from Ge et al. were included in this study. (D) Microbial richness per cancer type. (E) Spearman correlation of microbial richness between the two TMPs.

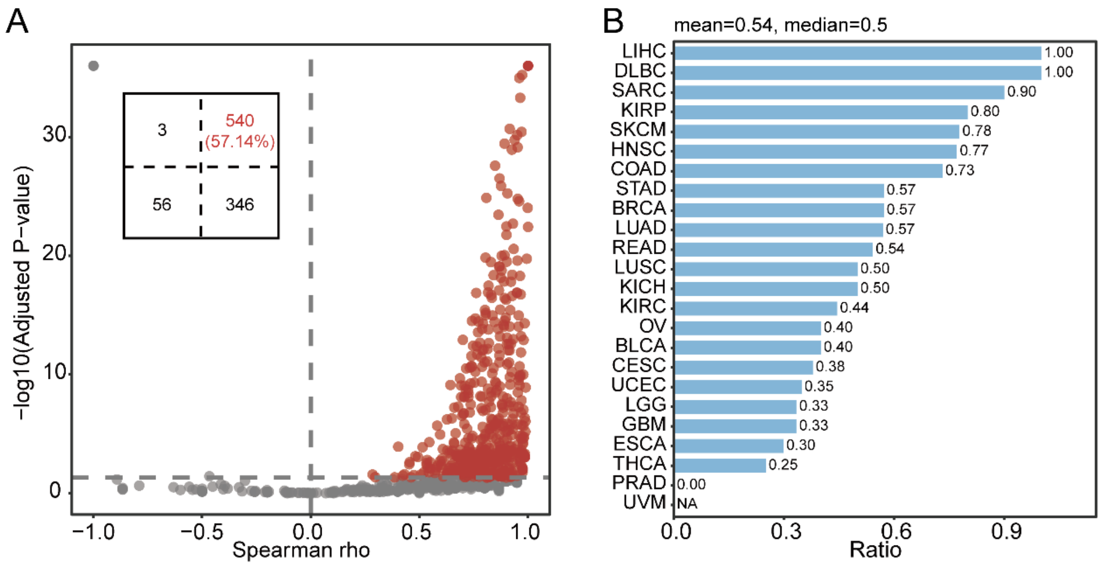

Figure S2. Spearman correlations for shared microbes with non-zero abundance in both TMPs in each cancer types. (A) Red points indicate a significant positive correlation (BH-adjusted $p < 0.05$, rho > 0), and gray points indicate non-significance positive associations. (B) Proportion of microbial species per cancer type showing a significant positive Spearman correlation. Samples from UVM were designated as ' not available (NA)' due to the absence of evaluable microbes.

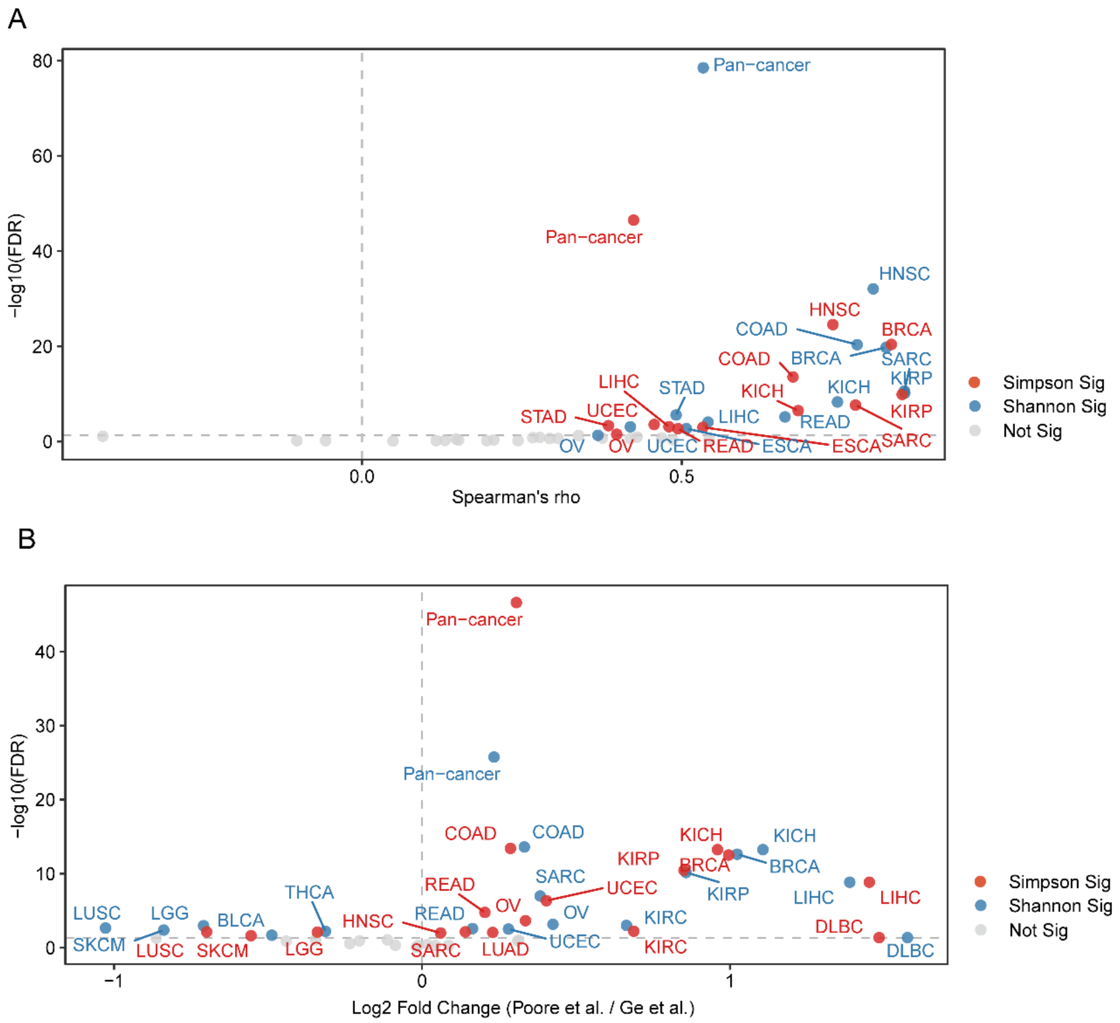

Figure S3. The consistency Shannon and Simpson indices for shared microbes in both TMPs in each cancer types and pan-cancer. (A) Spearman correlation analysis of Shannon and Simpson indices between the two TMPs. (B) Differential comparison of Shannon and Simpson indices between the two TMPs. Blue represents cancer types with significant differences in the Shannon index, red indicates those significant in the Simpson index, and gray denotes non-significant results.

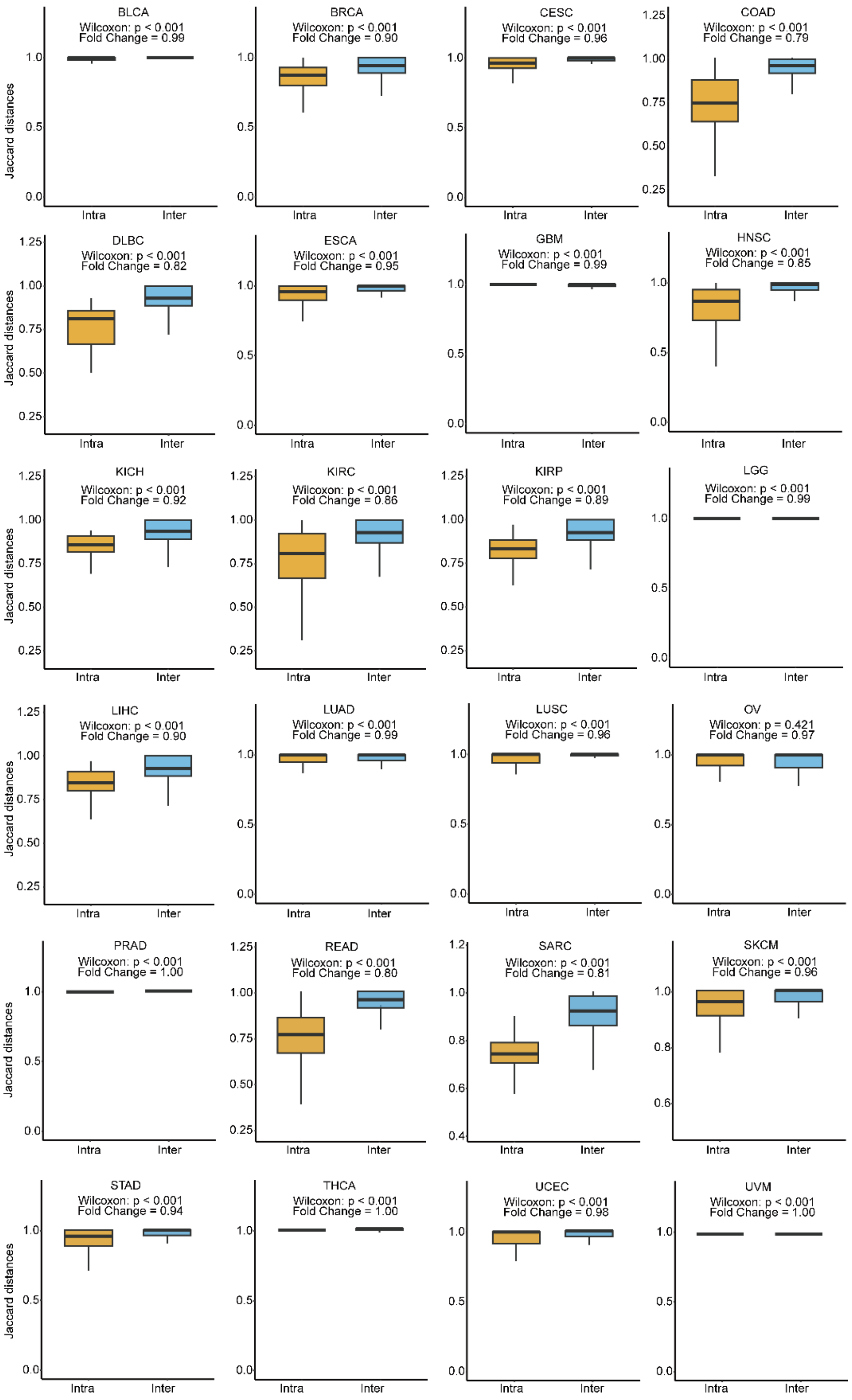

Figure S4. Comparison of Jaccard distances between Ge et al. and Poore et al. matched cancer types and unmatched cancer types. For each cancer type in Ge et al., compare its Jaccard distance to its matched type in Poore et al. versus its distances to all other (unmatched) types in Poore et al. The p-values were calculated using the Wilcoxon rank-sum test.

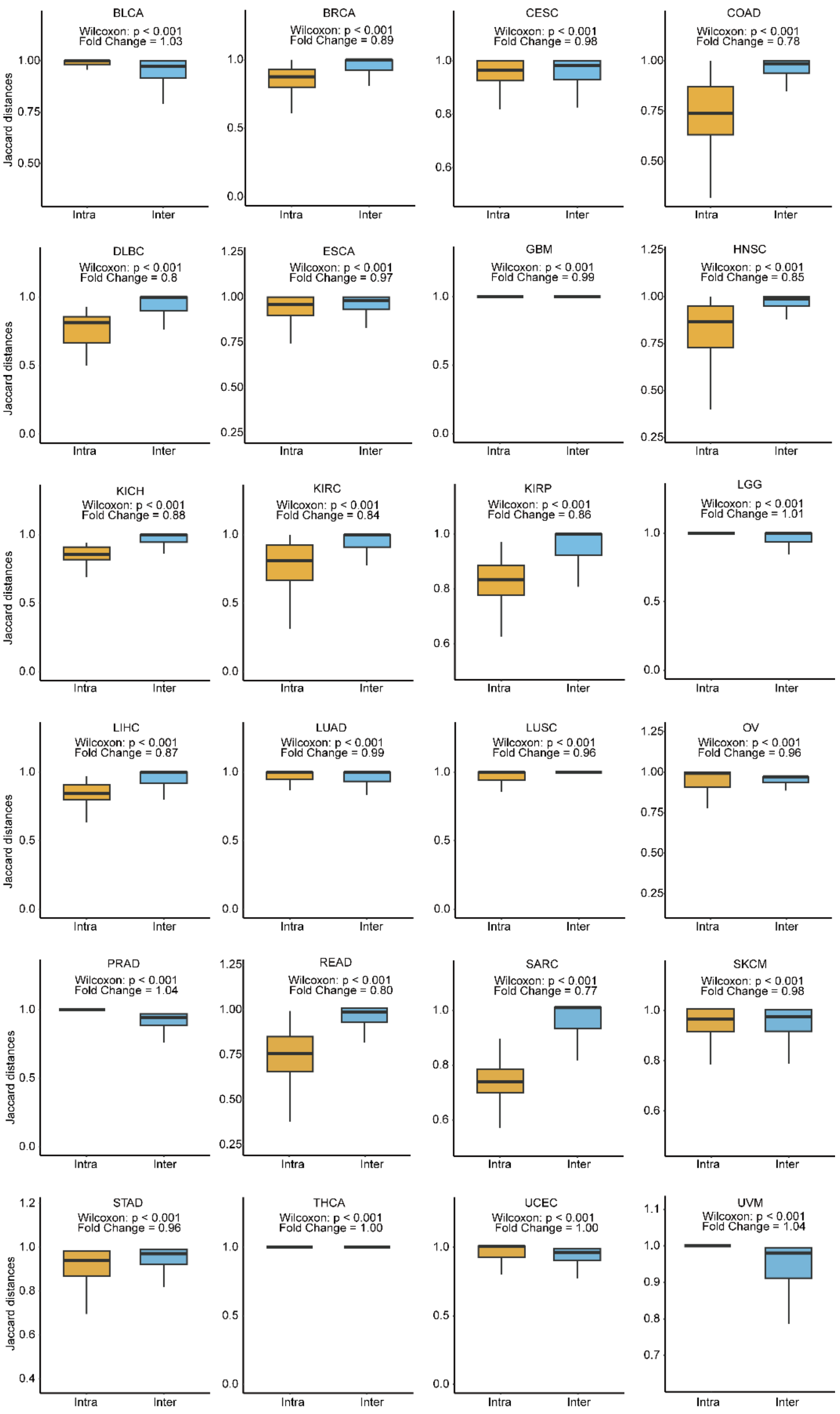

Figure S5. Comparison of Jaccard distances between Poore et al. and Ge et al. matched cancer types and unmatched cancer types. For each cancer type in Poore et al., compare its Jaccard distance to its matched type in Ge et al. versus its distances to all other (unmatched) types in Ge et al. The p-values were calculated using the Wilcoxon rank-sum test.

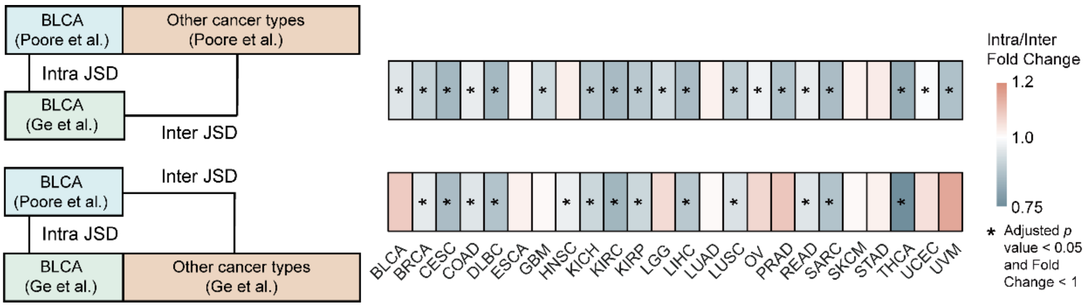

Figure S6. Comparison of intra-cancer versus inter-cancer Jensen-Shannon Divergence. The intra-cancer distance (comparing the same cancer type between TMPs, e.g., BLCA-Poore vs. BLCA-Ge) is compared to the distribution of inter-cancer distances (e.g., BLCA-Poore vs. all other cancers in Ge). P values were calculated using the Wilcoxon rank-sum test. Asterisks indicate significant differences (adjusted $p < 0.05$) where intra-distances are smaller than inter-distances.

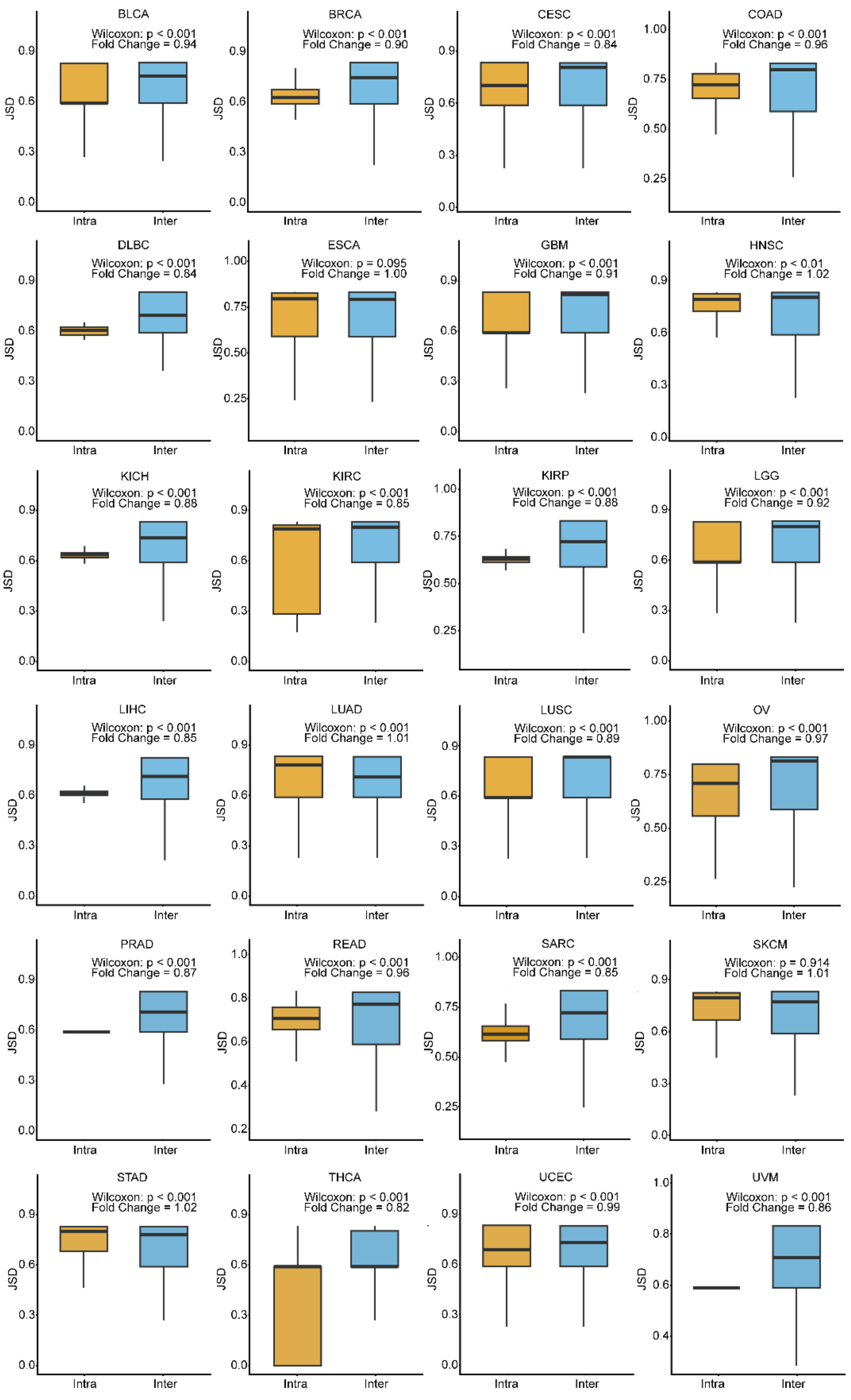

Figure S7. Comparison of Jensen-Shannon Divergence between Ge et al. and Poore et al. matched cancer types and unmatched cancer types. For each cancer type in Ge et al., compare its Jensen-Shannon Divergence to its matched type in Poore et al. versus its distances to all other (unmatched) types in Poore et al. The p-values were calculated using the Wilcoxon rank-sum test.

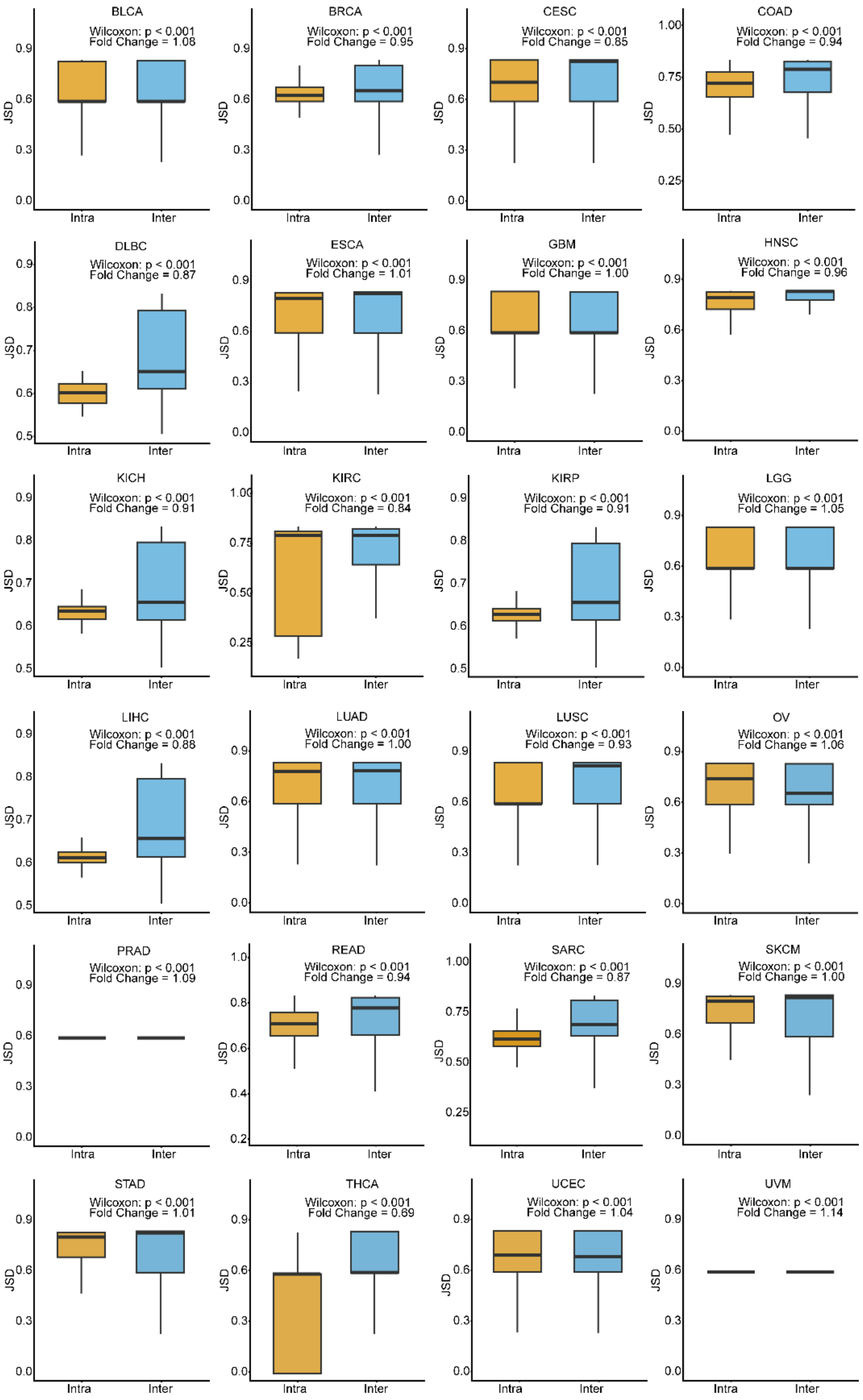

Figure S8. Comparison of Jensen-Shannon Divergence between Poore et al. and Ge et al. matched cancer types and unmatched cancer types. For each cancer type in Poore et al., compare its Jensen-Shannon Divergence to its matched type in Ge et al. versus its distances to all other (unmatched) types in Ge et al. The p-values were calculated using the Wilcoxon rank-sum test.

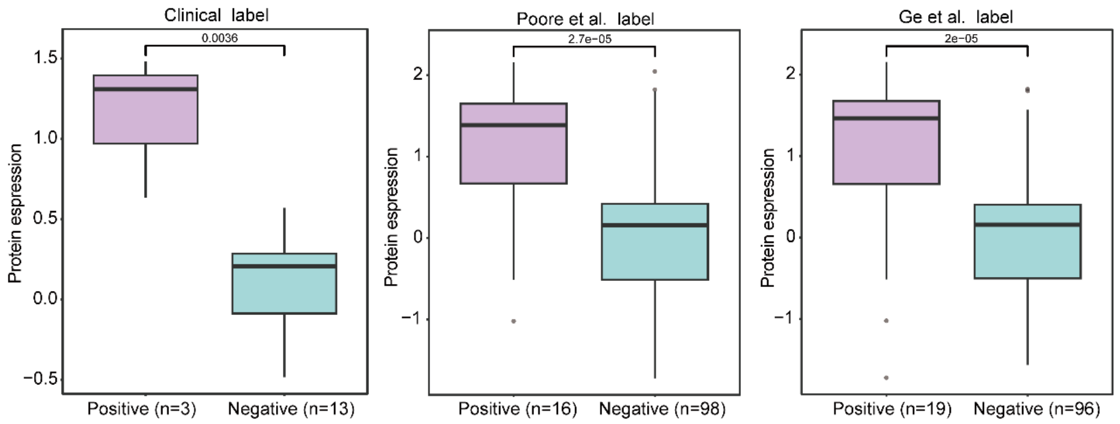

Figure S9. Comparison of P16INK4A expression in HPV positive and negative group based on different labels. The p-values were calculated using the Wilcoxon rank-sum test.

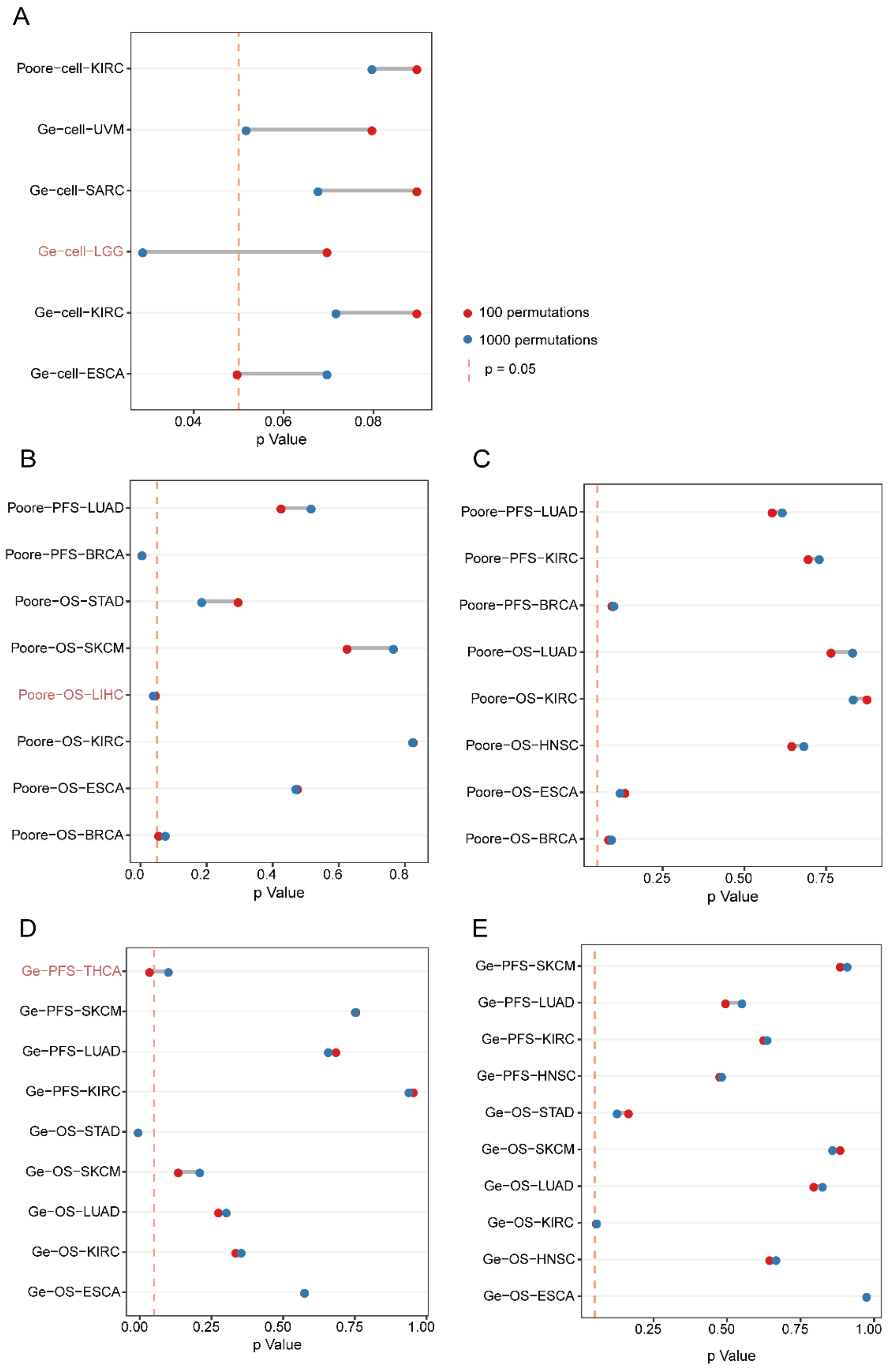

Figure S10. Comparison of results between 100 and 1,000 permutations. (A) Associations between microbial TMPs and cell types. (B, D) Survival associations based on microbial presence-absence data. (C, E) Survival associations based on microbial relative abundance. Only cancer types with positive events identified in the original TMPs were included in the permutation comparisons. The tests highlighted in red indicate inconsistent results, while the vast majority of tests demonstrate robustness.

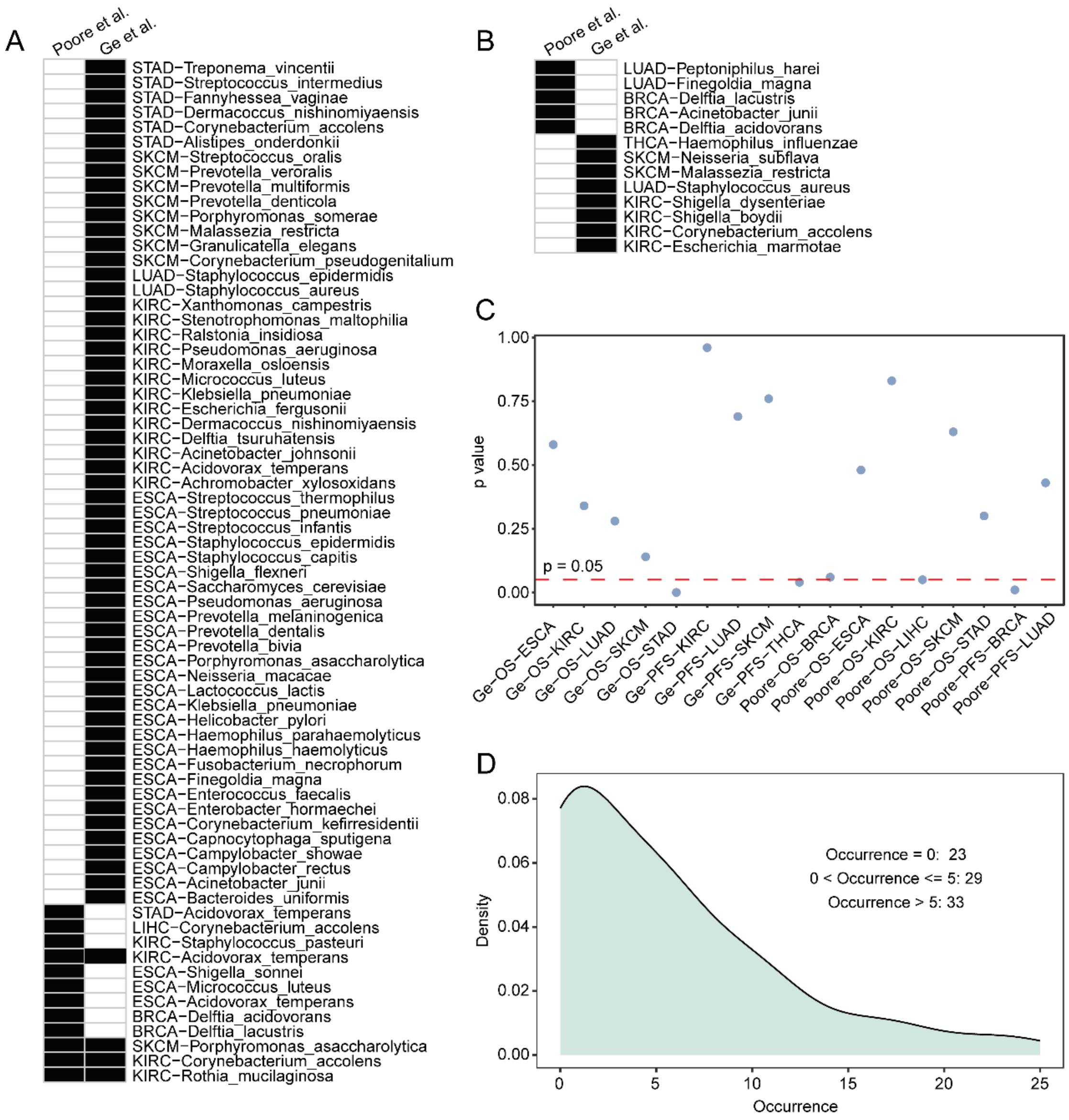

Figure S11. Permutation-based benchmarking of the clinical impact of current TMPs. The distribution and overlap microbes associated with OS (A) and PFS (B) across cancer types. (C) Distribution of OS- and PFS-associated microbes in permuted TMPs; p-values were derived from permutation outcomes. (D) The distribution of association recurrences across permutations.

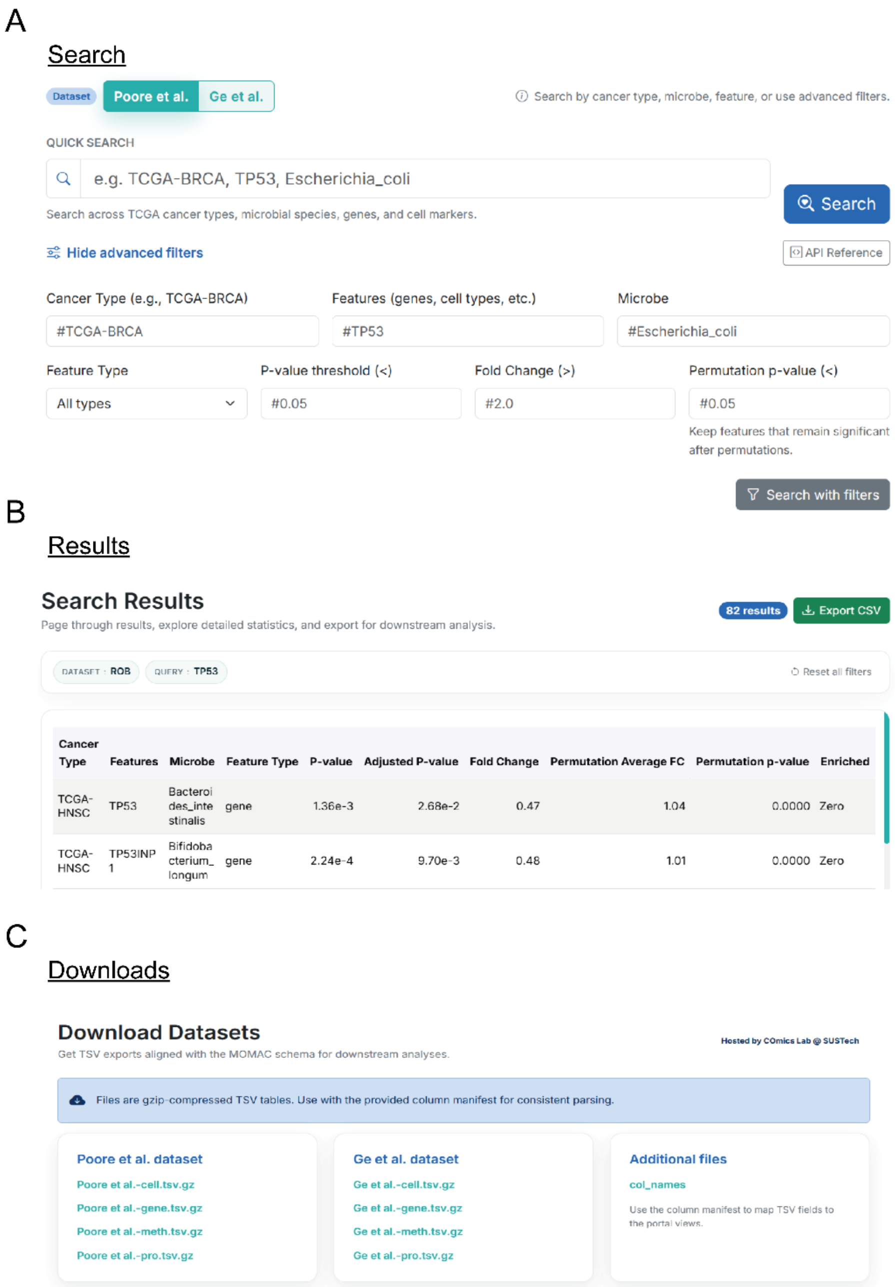

Figure S12. A comprehensive user guide for MOMAC2. MOMAC2 comprises three primary functionalities: (A) Search, (B) Results, and (C) Downloads. Users can perform multi-dimensional queries by specifying microbes, cell types, genes, methylation sites, or proteins. The interface provides a comprehensive summary of associations, including cancer types, raw and adjusted p-values, fold changes, as well as permutation-based fold changes and p-values with group enrichment details. For users interested in high-throughput or large-scale analyses, MOMAC2 supports the batch download of all association results, facilitating customized downstream filtering and integration via local scripts.

Table S01. Metadata of TCGA samples used in this study, related to Figure S1A and Figure S10.

Table S02. Species list used in this study from Poore et al., Ge et al. and intersections related to Figure S1B-C.

Table S03. TCGA-derived microbial profiles from Poore et al.

Table S04. TCGA-derived microbial profiles from Ge et al.

Table S05. The frequency of zero in each species, related to Figure 1A.

Table S06. Odd ratio and adjusted p value for Fisher's tests, related to Figure 1B-C.

Table S07. The Spearman correlation coefficients of the 231 shared species between the TMPs, related to Figure S2A-B.

Table S08. The Shannon and Simpson correlation coefficients of the 231 shared species between the TMPs, related to Figure S3.

Table S09. Jaccard distance, related to Figure 1D.

Table S10. Jensen–Shannon divergence, related to Figure S6-8.

Table S11. Clinical and TMPs detection for cancer-related microbes, related to Figure 1H.

Table S12. Distribution of MOMAC2, related to Figure 2B.

Table S13. Jaccard index at the species level, related to Figure 2C-D.

Table S14. Permutation results at the cancer and omics level, related to Figure 3.

Table S15. Public data and TMPs, related to Figure 4.

Table S16. The associations between HPV infection and host gene expression in HNSC based on two TMPs, clinical label, permuted label and public data, related to Figure 4A-D.

Table S17. Intersections in Table S14, related with Figure 4E-F.

Table S18. HPV-derived host gene expression in CESC.

Table S19. EBV-derived host gene expression in STAD.

Table S20. H. pylori-derived host gene expression in STAD.

Table S21. HBV-derived host gene expression in LIHC.

Table S22. *F. nucleatum*-derived host gene expression in COAD.

Table S23. The associations between tumor microbiome and OS, PFS, related to Figure S4A.

Table S24. Permutation-based benchmarking of the associations between tumor microbiome and OS, PFS, related to Figure S4B-C.

Table S25. Differential level distribution in MOMAC2, related to Figure 5B.

Table S26. Methylation-gene expression axis in MOMAC2, related to Figure 5C.

Table S27. Mediation analysis, related to Figure 5C.

Table S28. Differential gene expression of *Streptococcus anginosus* and CAL27 co-culture, related to Figure 5G.
